# Comparison of text messaging data collection vs face-to-face interviews for public health surveys: a cluster randomized crossover study of care-seeking for childhood pneumonia and diarrhoea in rural China

**DOI:** 10.7189/jogh.08.010802

**Published:** 2018-06

**Authors:** Michelle Helena van Velthoven, Wei Wang, Qiong Wu, Ye Li, Robert W Scherpbier, Xiaozhen Du, Li Chen, Yanfeng Zhang, Josip Car, Igor Rudan

**Affiliations:** 1 Global eHealth Unit, Department of Primary Care and Public Health, Imperial College London, London, United Kingdom; 2 Department of Paediatrics, Medical Sciences Division, University of Oxford, Oxford, United Kingdom; 3 Department of Integrated Early Childhood Development, Capital Institute of Paediatrics, Beijing, China; 4 Health and Nutrition, UNICEF China, Beijing, China; 5 Centre for Population Health Sciences (CePHaS), Lee Kong Chian School of Medicine, Nanyang Technological University, Singapore, Singapore; 6 Centre for Population Health Sciences and Global Health Academy, University of Edinburgh Medical School, Edinburgh, United Kingdom; * These authors contributed equally to this work.

## Abstract

**Background:**

To compare text messaging and face-to-face interviews to conduct a survey on childhood diarrhoea and pneumonia.

**Methods:**

Caregivers of young children able to send text messages in Zhao County in rural China were included in this crossover study. Villages (clusters) were randomized into two groups using the ratio 1:1.6 to account for an expected higher drop-out in group 2. In group 1, participants first completed the face-to-face and then text messaging survey; this order was reversed in group 2. We determined data equivalence of 17 questions that were answered by participants who were the same person in both surveys. For the text messaging survey, we assessed the overall and item response rate.

**Results:**

We included 1014 participants between 16 and 28 March 2013: 371 in 15 villages in group 1 and 643 in 27 villages in group 2. A total of 662 (65.3%) out of 1014 participants responded (first text message question) and a significantly higher proportion who did not respond were from rural areas (*P* = 0.005). Of 651 participants willing to participate, 356 (54.7%) completed the text messaging survey, which was marginally significantly different between the groups (*P* = 0.05). In total, 409 participants took part in both surveys: 183 in group 1 and 226 in group 2. There was a significantly higher proportion of caregivers from rural areas in Zhao County in the non-responder group compared to the responder group (*P* = 0.004). Kappas were substantial for six (0.61–0.80), moderate for two (0.58 and 0.60), and fair for three questions (0.31, 0.35 and 0.37). The proportion of agreement was >90% for five questions; 80.0%-90.0% for five questions; 70.0%, 65.0% and 45.5%. The remaining questions had too small numbers to calculate these values.

**Conclusions:**

This study shows that text messaging data collection produces data similar to data from face-to-face interviews in a middle-income setting, but the response rate was insufficient for use in public health surveys. Improving the response rate is important, because text message surveys could be of greater value in rural remote areas due to the cost-saving potential.

Childhood diarrhoea and pneumonia are the leading infectious causes of death in children under five globally [1]. Coverage of interventions that could prevent many deaths is too low in low- and middle-income countries [2,3]. Information on care-seeking is essential to implement interventions but is limited in these countries where health information systems are weak [4-6]. Household surveys are the primary source of child health indicators in low- and middle-income countries [7]. However, the current face-to-face surveys are costly and time-consuming to undertake and cannot be conducted on very large samples or frequently.

Costs are one of the biggest constraints for designing and implementing household surveys. Costs for field visits include personnel, transport, accommodation, equipment, consumables and other costs. Recruitment and training of interviewers and supervisors is labor-intensive, time-consuming and expensive [8]. Moreover, interviewers can have difficulty accessing households because of poor transportation, caregivers’ unavailability and security concerns. There are difficulties in gaining access to particular areas, for example during the wet season when roads are difficult or impossible to pass [9]. Caregivers often work outside home during day-time or are too busy to be interviewed. Moreover, some cultural customs prevent interviewers from visiting households. For example, in rural China people believe that it is not good for newborns and mothers to be visited by people within the first months after birth [10]. It is usually not feasible to conduct household surveys that include very large samples of participants. However, a large sample size is needed for disaggregated analysis by sex, age and socio-economic position [11] and for obtaining adequate denominators to support coverage measurement when the two-week point prevalence of events is very low [3]. Household surveys cannot often be conducted frequently. Regular household surveys carried out according to minimum standards are required to provide frequent data for programme monitoring [12].

In addition, these surveys have issues with interviewer variance and influence, privacy, confidentially and data quality control [13]. Most household surveys are sample surveys in which representative samples are preselected with each household having a known chance to be selected. In the Chinese study context, a list with names of children is obtained through routine health information systems. A common problem is the low quality of health information systems and lists of names of people are often incomplete and inaccurate [4,5]. Interviewers can introduce bias in household surveys. Demographic and Health Surveys and Multiple Indicator Cluster Surveys have minimum requirements for selecting interviewers. This means that interviewers need at least a high school diploma and cannot directly be involved in the management or provision of health services to avoid potential conflict of interest [7]. In addition, all interviewers are trained according to the survey protocol and evaluated before conducting field work. However, interviewer bias cannot be completely avoided, because interviewers can influence interviews and introduce response bias and socially desired answers. Additionally, concerns have been raised about data quality issues and interviewers fabricating data in low- and middle-income countries [14]. Experiences indicate that interviewers sometimes modify children’s age, for example by transferring children to an age group of over five years, to exclude them from the survey sample and thereby reduce their workload [7].

Self-administered surveys based on mobile phones offer a promising alternative to the current interviewer-administered household surveys [15]. By the end of 2016, there were an estimated 7.5 billion mobile phone subscriptions [16]. Text messaging is popular and user-friendly, because of its immediacy, low costs and non-intrusiveness [17]. Text messaging could be used for real-time data collection at the household level and has the potential for scale-up at low cost in different settings [13]. Text messaging can overcome geographical barriers and be used to contact people who are difficult to reach [18].

However, the mode of data collection can have effects on data quality [19]. While self-administered surveys are not biased by an interviewer and can be completed at a private location, these surveys often have a lower response rate than interviewer-administered surveys [19]. There is a greater risk that respondents misinterpret questions in self-administered surveys and thus these surveys require excellent questionnaire design [20]. Furthermore, text messaging data collection faces several challenges including coverage of networks, access to mobile phones, payment, and usage of text messaging [21].

To inform this study on current evidence for use of text messaging for health data collection, one reviewer searched the English literature (MV). One reviewer (QW) searched Chinese databases (Wanfang Data and China National Knowledge Infrastructure) but found no studies. Relevant search terms ((phon*, mobil* OR mHealth OR “m health” OR m-health OR eHealth OR telemedicine [MeSH]) AND (data OR information OR collect* OR gather* OR obtain* OR monitor* OR data collection [MeSH])) were used. Both English language databases (The Cochrane Central Register of Controlled Trials, PubMed, EMBASE, World Health Organization Global Health Library regional index, PsycINFO, Web of Science, MobileActive and Royal Tropical Institute “KIT” Information Portal mHealth in Low-Resource Settings). This review excluded studies where health workers were using mobile phones to collect information and our studies that were published after the review was conducted [22,23].

A total of 19 studies reported in 21 papers [24-44] evaluated use of text messaging for collection of health-related information, which showed to be acceptable to participants [28,30,33,34,43,44] and easy to use [36, 37] (Table S1, S2 and S3 in **Online Supplementary Document(Online Supplementary Document)**). The completion rate varied widely from 15% [41] to 100% [37]. Eight out of 10 studies that compared text messaging with other forms of data collection assessed data equivalence [26,28,34-36,39,41,42]. Data equivalence was high when comparing text messaging to: telephone interviews on influenza vaccination [41]; telephone interviews and health visitor interviews on infant feeding [28]; telephone interviews for 1-week and 1-month recall of low back pain [34]; an Internet survey and paper survey on sexual behaviour [35]; verbal assessment of pain in presence of physician [36]; a baseline Internet survey on usual quantity of drinks [39]; and paper cards on satisfaction of primary care consultation [42]. Data equivalence was low for the 1-year recall of low back pain by telephone interviews [34], and for a baseline Internet survey about the number of drinks [39] compared with data collected via text messaging. However, many studies did not compare data collection methods, used small samples, or had a non-randomized study design. Moreover, few studies took place in a low- or middle-income country [24,33,37]. No studies were found on using text messaging for household data collection in LMIC. We conducted two feasibility studies on the collection of information related to infant feeding via text messaging in China [22,23].

To address these gaps in the literature, build on our previous work and inform the use of text messaging data collection in LMIC, we aimed to assess the use of text messaging for a survey on childhood diarrhoea and pneumonia in rural China by addressing the following objectives. First, to assess for the text messaging survey: (i) the response rate; (ii) differences between responders and non-responders; and (iii) the error proportion. Second, to compare text messaging to the standard face-to-face interview method in terms of: (i) data equivalence; (ii) the amount of information in responses; and (iii) reasons for differences in responses. A cluster randomized design was used as participants could not be randomized on an individual level for organizational reasons.

## METHODS

### Design

This study was part of a larger project described elsewhere, of which overall aim was to explore the application of mHealth-based collection of information relevant to childhood diarrhoea and pneumonia in rural China [45]. We compared a face-to-face survey (reference standard) to a text messaging survey (novel method). We randomised villages (clusters) into two groups. Participants were caregivers of young children. Participants either completed the face-to-face survey first and then the text messaging survey (group 1) or the other way around (group 2). We used a crossover design to allow assessment of whether differences were mode related.

### Study setting and sample

The study took place in Zhao County which is located in Hebei Province, situated in the northern part of the North China Plain with an area of 190 000 km^2^ (for comparison, the size of the United Kingdom is 245 000 km^2^), bordering the capital Beijing. Zhao County has a total population of 571 000, with 518 000 people (90.7%) living in rural areas. Zhao County covers an area of 675 km^2^ and is located 40 km south of Shijiazhuang City. The socioeconomic development of Zhao County is similar to Hebei Province, similar to the national average. The annual per capita net income was ¥ 6464 (about US$ 1026) for residents in Zhao County, ¥ 5958 (about US$ 946) for residents of Hebei Province, and the national average was ¥ 5919 (about US$ 946) in 2010 [46,47]. The female illiteracy rate is low (3.8%) and the main ethnic group is Han (99.9%) (data from 2010 provided by the Zhao County Statistics Bureau, unpublished). A survey among 1601 caregivers of young children in Zhao County showed that 99.4% of households had at least one mobile phone. Moreover, 61.2% of the households owned computers, with 54.8% having access to Internet (QW, personal communication). Zhao County has four hospitals at county level: (i) a public general hospital; (ii) a public maternal and child health hospital; (iii) a public traditional Chinese medicine hospital; and (iv) a private general hospital. Each of the 16 townships has a public township hospital and all the 281 villages have a village clinic. Village clinics are often privately-owned by village doctors who receive small subsidies from the government for providing public health services. Village doctors live in the communities they serve and have a good relationship with villagers. Village doctors provide primary health care at village level and are trained and supervised by staff at township and county-level. Education and training of village doctors varies, but usually they have at least primary school or junior high school and short basic medical training [48,49]. The study took place in one township, Zhaozhou Township, which has both a semi-urban and rural area and 46 villages.

Village doctors gathered caregivers in their village clinics where interviewers recruited them. Views of village doctors and caregivers on participation in this study are presented elsewhere [50]. Clusters were included if the village doctor was present and willing to participate as without their help caregivers could not be recruited for the study. The participant inclusion criteria were: had a child in the family younger than five years (the youngest child if there was more than one child), have access and able to use a mobile phone, able to text message, and provided consent. Family members, including grandparents, often take care of the children in China and were included to maximize the recruitment rate.

We aimed to recruit a reasonable number of caregivers in the study setting and generate parameters for sample size calculations in future studies. It was not possible to conduct an accurate sample size calculation for the crossover study, because it was a feasibility study and precise estimates from previous research that could inform a calculation were unavailable. The local township hospital provided a list of 4170 names of children younger than five years who lived in Zhaozhou Township (almost all women deliver in hospitals in China). However, the accuracy of this list of names was not known and based on previous research experiences we expected that a significant number of children on the list were not living in the villages of the list of names.

### Randomization

**Figure 1:**
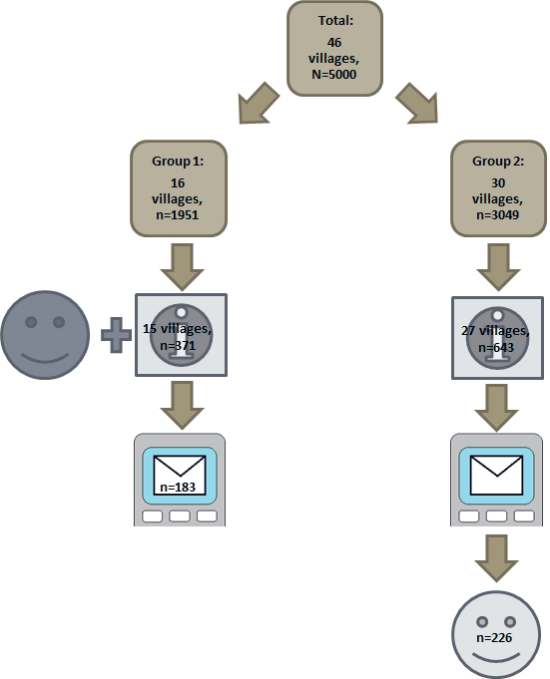
Flow diagram of study participants.

The local health officials provided consent before randomization. Our statistician (WW) randomized villages using SAS version 9.2 [51]. **Figure 1** shows that the 46 villages were randomized as follows: 16 villages (with 1600 children) into group 1 and 30 villages (with 2570 children) into group 2. The villages were ranked based on the size of their under-five population into three strata of 15, 15 and 16 villages each. An independent statistician provided a list of random numbers to determine the strata that had 16 villages. WW used block-randomization with a ratio of 1:1.6 to allocate a larger proportion of participants to group 2 and to account for the expected higher drop-out (participants had to come back to the village clinic for the face-to-face survey). WW enrolled the villages and assigned them to group 1 and group 2. Differences between group 1 and 2 were the sequence of the face-to-face and text messaging surveys and group 2 were asked about their reasons for differences in responses.

### Questionnaires

The face-to-face questionnaire included questions on identification (including mobile phone number) and demographics (about 30 questions), and 17 on care-seeking for childhood diarrhoea and pneumonia (Table S4 in **Online Supplementary Document(Online Supplementary Document)**). These questions were taken from the World Health Organization Maternal, Newborn and Child Health Household survey that has been used in China since 2010 (2009 version, unpublished). In addition, the questionnaire had questions on mobile phone usage developed by the research team.

A subset of 17 questions on care-seeking was also administered via text messaging and compared between the methods (Table S4 in **Online Supplementary Document(Online Supplementary Document)**). Moderate changes had to be made to the face-to-face questionnaire to make it appropriate for a text messaging survey format. It was aimed to develop a text message questionnaire in which the text message questions were interpreted by caregivers in a similar way as the face-to-face questions. Cognitive interviewing and usability testing was an adequate research strategy for this purpose. The questions are reported by the number of the text message in which they were asked (text message 4 to 20) [45]. The text messaging survey had a total of 19 questions. In addition to the 17 comparison questions, first we asked caregivers to confirm that they were willing to participate (text message 2) and about the relationship between the caregiver and the child (text message 3). In addition to these questions, text message 1 greeted and text message 21 thanked participants.

The text message questions were developed in the following way. First, we converted the face-to-face survey questions into questions suitable for text messages. Second, we tested the questions using cognitive interview techniques to check understanding of the meaning of the questions [52]. Third, we conducted a pilot in which we sent text messages to 217 caregivers from another township in Zhao County (Shahedian Township) and revised the questions based on the results [45].

### Recruitment and data collection

The study took place between 16 and 28 March 2013. We asked village doctors to gather all caregivers of young children in their village clinic.

In group 1, trained interviewers obtained informed consent and administered the entire face-to-face questionnaire. After one day, we sent the text messages for two days during which participants had to complete the survey, with 2 reminder text messages if they did not respond.

**Figure 2:**
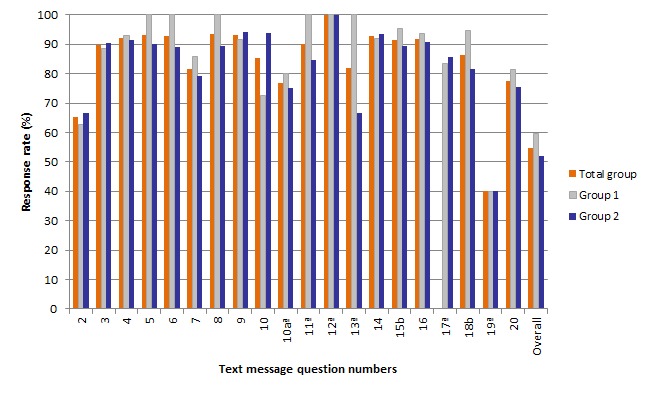
Item response rate (%) and completion rate for the total group. (N = 1014), group 1 (n = 371) and group 2 (n = 643).

In group 2, interviewers obtained informed consent and administered the face-to-face questionnaire, but did not ask the questions about care-seeking (see **Figure 2**). Then we sent the text messages. Participants who responded to text message 4 (first question that was compared between the methods) were invited to visit the village clinic one for the face-to-face questions on care-seeking one day after finishing the text messaging survey (similar recall period as in group 1). Directly after completing the face-to-face questions, participants who did not give the same response in both surveys were asked about their reasons for this.

Interviewers used smartphones to record responses in the face-to-face interview [53]. Participants received a towel (worth ¥ 5, about US$0.82, £ 0.52, € 0.62) for completing the face-to-face survey. One researcher (YL) manually sent and received text messages using a Chinese text messaging system (Shāng jī bǎo 商机宝). A second trained researcher checked the text messages that were sent out to prevent errors. We paid participants ¥ 1 back for text message costs (sending one text message in China costs ¥ 0.10) and ¥ 5 when they completed the text message survey (thus participants received ¥ 1 when they sent at least one text message and ¥ 6 when they answered all questions). All data were wirelessly and securely transferred into an Excel database via an internet server and could only be accessed by the researchers involved in this study.

### Outcomes and analysis

SPSS version 16.0 [54] and SAS version 9.2 [51] were used for analysis. We assessed the item response rate as the proportion of participants who participated and responded to individual questions and completion rate as the proportion of participants who participated and completed the survey. We compared participants who responded to those who did not respond to text message 2 (responders vs non-responders) and participants who completed the survey to those who did not complete the survey (completers vs non-completers) in group 1. Characteristics were compared using the Pearson χ^2^ test and Fisher Exact test for nominal variables and Mann-Whitney U/ Wilcoxon W (MWU/WW) test for not normally distributed continuous variables and ordinal variables. We calculated the error proportion of the text messaging method, which was evaluated by incorrect text message questions that were sent to participants and incorrect text message answers that were received from participants. We counted the reasons for differences in responses.

We calculated kappa values and the proportion of agreement for data equivalence and the amount of information. For these analyses, we included the participants who responded to text message 4 and were the same caregivers participating in both surveys. We calculated the results for group 1 and 2 together and for the two groups separately. We present data equivalence for 15 nominal questions of which 10 were dichotomous (text message 4, 5, 6, 7, 8, 11, 14, 15, 16, 18) and 5 were non-dichotomous (text message 12, 13, 17, 19, 20). We present Cicchetti-Allison and Fleiss-Cohen weighted kappa (two commonly used kappa weights) for two ordinal questions (text message 9 and 10a) [55]. Kappa values have the following meaning: <0.00 poor; 0.00-0.20 slight; 0.21-0.40 fair; 0.41-0.60 moderate; 0.61-0.80 substantial; and 0.81-1.00 almost perfect [56].

### Ethics approval

The Ethical Committee of the Capital Institute of Pediatrics in Beijing provided ethical approval (No. SHERLL 2013009). All participants provided written informed consent for both the face-to-face and text messaging survey prior to their inclusion in the study. We anonymized participant identifiable information for data analysis and reporting.

## RESULTS

### Participants

**Figure 1** shows the flow diagram of participants. Of the 4170 children on list of names, we were able to recruit 1026 caregivers in 42 villages and we included 1014 participants: 371 in 15 villages in group 1 and 643 in 27 villages in group 2 (Table S5 in **Online Supplementary Document(Online Supplementary Document)**).

The remaining 3144 caregivers of children (4170 minus 1026) could not be recruited, because they were not present in the village, the list of names was incorrect, they did not meet the inclusion criteria or village doctors were unavailable (in one village in group 1 and in three villages in group 2). The specific numbers for each reason were not possible to register because the accuracy of the name list was unknown. We excluded 12 caregivers for the following reasons: the child of one caregiver just reached the age of five; we did not send text messages to three caregivers in group 1 by an administrative mistake, and we could not identify which child belonged to the text message responses for eight caregivers.

Of 1014 included participants, there were 796 mothers (78.5%), 141 fathers (13.9%), 53 grandmothers (5.2%), 22 grandfathers (2.1%) and 2 aunts (0.2%). Out of 16 characteristics that we had available, 7 had significant differences between the groups (**Table 1**). In group 1 there were more children with an urban Hukou (*P* < 0.001), a higher proportion of participants were mothers (*P* = 0.003), a higher proportion of participants were the primary caregiver of the child (*P* = 0.001), mothers (*P* = 0.03) and fathers (*P* = 0.007) had a higher education level, fathers had a higher median number of years of education (*P* = 0.046), the family income was lower (*P* = 0.03) compared to group 2. Out of the 13 indicators related to mobile phone usage, 1 had a significant difference between the groups (Table S6 in **Online Supplementary Document(Online Supplementary Document)**).

**Table 1:** Characteristics of children under-five and caregivers

Variables	Total (N = 1014)	Group 1 (n = 371)	Group 2 (n = 643)	Comparison	
Statistics*	***P*-value**
**Gender, n (%):**				χ^2^ = 0.03	0.87
Boy	561 (55.3)	204 (55.0)	357 (55.5)		
Girl	453 (44.7)	167 (45.0)	286 (44.5)		
**Age child groups, n (%):**				MWU/WW z = -0.41	0.68
0-11 months	212 (20.9)	74 (19.9)	138 (21.5)		
12-23 months	279 (27.5)	112 (30.2)	167 (26.0)		
24-59 months	523 (51.6)	185 (49.9)	338 (52.5)		
**Number of children, n (%):**				MWU/WW z = -0.49	0.62
1	470 (46.4)	174 (46.9)	296 (46.0)		
2	521 (51.4)	192 (51.8)	329 (51.2)		
3	21 (2.1)	4 (1.1)	17 (2.6)		
4	2 (0.1)	1 (0.2)	1 (0.2)		
**Mother’s age in years, median (Q1-Q3)**	28 (26-31)	28 (26-31)	28 (26-32)	MWU/WW z = -0.30	0.76
**Mother’s education level, median (Q1-Q3)†**	3 (3-3)	3 (3-4)	3 (3-3)	MWU/WW z = -2.26	0.03
**Mother’s number of years of education, median (Q1-Q3)**	9 (9-9)	9 (9-11)	9 (9-9)	MWU/WW z = -1.41	0.16
**Mother’s occupation, n (%):**				Fisher exact test	0.33
Home	496 (48.9)	171 (46.1)	325 (50.5)		
Work	515 (50.8)	199 (53.6)	316 (49.1)		
Do not know	3 (0.3)	1 (0.3)	2 (0.4)		
**Father’s age in years, median (Q1-Q3)**	29 (26-32)	29 (27-32)	29 (26-32)	MWU/WWz-0.15	0.88
**Father’s education level, median (Q1-Q3)†**	3 (3-3)	3 (3-4)	3 (3-3)	MWU/WW z = -2.68	0.007‡
**Father’s number of years of education, median (Q1-Q3)**	9 (9-9)	9 (9-12)	9 (9-9)	MWU/WW z = -2.00	0.046‡
**Father’s occupation, n (%):**				Fisher exact test	0.51
Home	12 (1.2)	6 (1.6)	6 (0.9)		
Work	998 (98.4)	363 (97.8)	635 (98.8)		
Do not know	4 (0.4)	2 (0.6)	2 (0.3)		
**Relationship to the child, n (%):**				Fisher exact test	0.003‡
Mother	796 (78.5)	300 (80.9)	496 (77.1)		
Father	141 (13.9)	58 (15.6)	83 (12.9)		
Grandmother	53 (5.2)	9 (2.4)	44 (6.8)		
Grandfather	22 (2.2)	4 (1.1)	18 (2.8)		
Other§	2 (0.2)	0 (0.0)	2 (0.4)		
**Participant is primary caregiver, n (%):**				χ2 = 14.87	0.001‡
Yes	687 (67.8)	279 (75.2)	408 (63.5)		
No	327 (32.2)	92 (24.8)	235 (36.5)		
**Registered as urban or rural, n** (%):				χ^2^ = 15.96	<0.001‡
Urban	55 (5.4)	34 (9.2)	21 (3.3)		
Rural	959 (94.6)	337 (90.8)	622 (96.7)		
**Family net income in last year in ¥, median (Q1-Q3)**¶	25 000 (20,000- 40 000)	20 000 (15,000- 35 000)	25 000 (20,000- 40 000)	MWU/WW z = -2.24	0.03‡
Do not know family income, n (%)	252 (24.9)	104 (28.0)	148 (23.0)		
**Family living expenses in the last year in ¥, median (Q1-Q3)**	20 000 (10,000- 20 000)	20 000 (10,000- 20 000)	20 000 (10,000- 20 000)	MWU/WW z = -0.55	0.58
Do not know family living expenses, n (%)	209 (20.6)	84 (22.6)	125 (19.4)		

*χ^2^-test, Mann-Whitney U/Wilcoxon W (MWU/WW), quartile (Q), z-score (z).

†3 = junior high school, 4 = senior high school/technical school.

‡*P* < 0.05.

§Child’s aunt (wife of brother of father), child’s aunt (wife of brother of mother).

¶n = 646 for total group: n = 248 for group 1 and n = 398 for group 2.

### Response rate

Of 1014 participants, 65.3% (n = 662) responded to text message 2 (asking about willingness to participate). Eleven participants responded that they were unwilling to participate and 651 were willing to participate (**Figure 2** and Table S7 and S8 in **Online Supplementary Document(Online Supplementary Document)**). The item response rates were only significantly different between the groups for two questions (text message 15 about the child having an illness with a cough, *P* = 0.03 and text message 18 about seeking care for a child with a fever or cough, *P* = 0.049)out of the total of 19 text message questions. Of the 651 participants willing to participate, 54.7% (n = 356) completed the text message survey, which was marginally significantly higher in group 1 (59.8%, n = 137) than group 2 (51.9%, n = 219) (*P* = 0.05).

### Characteristics of responders vs non-responders

There was a significantly higher proportion of caregivers from rural areas in Zhao County in the non-responder group compared to the responder group (*P* = 0.004). In addition, the median number of text messages received was significantly lower in the non-responder group compared to the responder group (*P* = 0.046) (Table S9 in **Online Supplementary Document(Online Supplementary Document)**). There were more children with diarrhoea in the non-completers group (*P* = 0.03) compared to the completers group (Table S10 in **Online Supplementary Document(Online Supplementary Document)**).

### Error proportion

**Figure 3:**
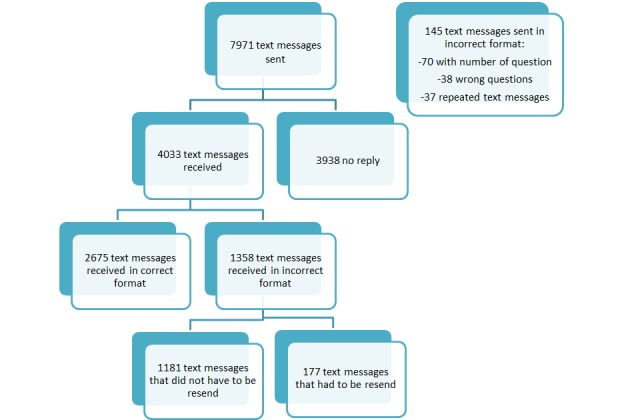
Incorrect text messages sent.

We manually sent a total of 7971 text messages of which 145 (1.8%) were in an incorrect format by mistake: 70 with an additional number (minor editing error), 38 wrong questions and 37 repeated questions. We received a total of 4033 text message replies from participants of which 1358 text messages (33.7%) were in an incorrect format and had to be assessed (**Figure 3**). After the assessment, we clarified the meaning of 1181 text messages (29.3%) and we did not have to send another text message to follow these responses up. For the remaining 177 text messages (4.4%), we could not clarify the meaning of the text messages and we had to resent our text message question (Figure S1 in **Online Supplementary Document(Online Supplementary Document)**). This process of resending text messages involved a total of 116 out of 662 participants responding to text message 2 (17.5%).

### Data equivalence

#### Total group

Kappa values were moderate or substantial for most of the 15 nominal dichotomous questions. However, for questions answered by a small number of participants, kappa was fair (text message 6 and 11, for which the 95% CI also overlaps) or could not be calculated (text message 5 and 7). The proportion of agreement was as follows for 10 nominal dichotomous questions: >90% for five questions; between 80.0% and 90.0% for four questions; and 70.0% for one question. Out of five non-dichotomous questions, the proportion of agreement and kappa could not be calculated for four questions (text message 12, 13 19 and 20) that had fewer than 10 participants (**Table 2** for total group, **Table 3** for group 1 and 2, and Table S11-S15 in **Online Supplementary Document(Online Supplementary Document)**). Weighted kappa values were substantial for Cicchetti-Allison and moderate for Fleiss-Cohen for text message 9. Both weighted kappa values were fair for text message 10a (**Table 4** and Table S16 and S173 in **Online Supplementary Document(Online Supplementary Document)**).

**Table 2:** Nominal questions, agreement and kappa coefficients for total group (N = 409)

Text message: brief content	Total group				
**No. of pairs**	**% agreement**	**Kappa**	**95% CI**	***P*-value**	
4: child had diarrhoea	409	96.8	0.76	0.64-0.89	<0.001‡	
5: child had blood in stools	21	100.0*	–	–	–	
6: child drank Oral Rehydration Salts	19	84.2	0.31	-0.27-0.90	0.16	
7: child drank recommended fluids	16	87.5*	–	–	–	
8: child drank other fluids	16	87.5	0.74	0.41-1.00	0.002‡	
11: sought care for diarrhoea	10	70.0	0.35	-0.24-0.94	0.26	
12: why care was not sought for diarrhoea†	2	2/2 agree*	–	–	–	
13: where care was sought for diarrhoea†	4	3/4 agree*	–	–	–	
14: child had fever	365	95.1	0.71	0.59-0.84	<0.001‡	
15: child had illness with cough	332	88.9	0.70	0.61-0.79	<0.001‡	
16: child breathed fast or difficultly	308	97.1	0.65	0.44-0.86	<0.001‡	
17: cause of fast or difficult breathing†	8	6/8 agree*	0.58	0.16-1.00	0.03‡	
18: sought care for child during fever or cough	55	94.5	0.77	0.52-1.00	<0.001‡	
19: why care was not sought for fever or cough†	2	2/2 agree*	–	–	–	
20: where care was sought for fever or cough†	40	65.0*	–	–	–	

CI – confidence interval

*Kappa cannot be calculated.

†Non-dichotomous.

‡*P* < 0.05.

**Table 3:** Nominal questions, agreement and kappa coefficients comparison between group 1 (n = 183) and group 2 (n = 226)

Text message: brief content	Group 1			Group 2		
**No. of pairs**	**Kappa or agreement**	**95% CI**	**No. of pairs**	**Kappa or agreement**	**95% CI**
4: child had diarrhoea	183	0.90	0.77-1.00	226	0.68	0.50-0.86
5: child had blood in stools	10	10/10 agree*	–	11	11/11 agree*	–
6: child drank Oral Rehydration Salts	10	0.41 (8/10 agree)	-0.18-1.00	9	8/9 agree*	–
7: child drank recommended fluids	9	7/9 agree*	–	7	7/7 agree*	–
8: child drank other fluids	9	0.57 (7/9 agree)	0.10-1.00	7	7/7 agree*	–
11: sought care for diarrhoea	5	4/5 agree*	–	5	3/5 agree*	–
12: why care was not sought for diarrhoea†	0	–	–	2	2/2 agree*	–
13: where care was sought for diarrhoea†	4	3/4 agree*	–	0	–	–
14: child had fever	164	0.77	0.61-0.93	201	0.66	0.47-0.84
15: child had illness with cough	156	0.66	0.53-0.79	176	0.74	0.62-0.86
16: child breathed fast or difficultly	146	0.70	0.42-0.98	162	0.60	0.28-0.92
17: cause of fast or difficult breathing†	5	0.38 (3/5 agree)	-0.21-0.96	3	3/3 agree*	–
18: sought care for child during fever or cough	31	0.71	0.34-1.00	24	0.83	0.52-1.00
19: why care was not sought for fever or cough†	1	1/1 agree*	–	1	1/1 agree*	–
20: where care was sought for fever or cough†	23	13/23 agree*	–	17	13/17 agree*	–

*Kappa cannot be calculated.

†Non-dichotomous.

**Table 4:** Ordinal questions, agreement and weighted kappa coefficients for total group (N = 409) and comparison between group 1 (n = 183) and group 2 (n = 226)

Text message: brief content	Total group					Group 1			Group 2		
**No. of pairs**	**% agreement**	**Cicchetti-Allison and Fleiss-Cohen kappa**	**95% CI**	***P*-value**	**No. of pairs**	**Cicchetti-Allison and Fleiss-Cohen kappa**	**95%CI**	**No. of pairs**	**Cicchetti-Allison and Fleiss-Cohen kappa**	**95% CI**
9: how much did child drink during diarrhoea	14	85.7	0.66, 0.60	0.23-1.00, 0.07-1.00	<0.001, 0.01†	8	0.53, 0.44	0.005-1.00, -0.18-1.00	6	1.00, 1.00	1.00-1.00, 1.00-1.00
10a: how much did child eat during diarrhoea	11	45.5	0.37, 0.37	0.05-0.70, -0.08-0.83	0.02†, 0.09	5	0.70 0.83	0.34-1.00, 0.58-1.00	6	0.13, 0.05	-0.10-0.36, -0.26-0.36
10a: how much did child eat during diarrhoea*	6	4/6 agree	0.64, 0.74	0.21-1.00, 0.35-1.00	0.03, 0.049†	–	–	–	–	–	–
10: child had been introduced to complementary foods	12	8/12 agree	–	–	–	–	–	–	–	–	–

CI – confidence interval

*We excluded children whose caregivers said that they had not been introduced to complementary feeding by either face-to-face interview or text messaging.

†*P* < 0.05.

#### Comparison between group 1 and 2

Five nominal questions had overlapping 95% confidence interval (CI) (text message 4, 14, 15, 16 and 18). Eight questions had too small number of participants to calculate 95% CIs: two questions had perfect agreement (text message 5 and 19) and six questions had fairly similar agreement (text message 6, 7, 8, 11, 17 and 20). The remaining two questions could not be compared (**Table 3**). For text message 9, weighted kappa values were moderate for group 1 but perfect for group 2. For text message 10a, weighted kappa was substantial for Cicchetti-Allison kappa and almost perfect for Fleiss-Cohen kappa in group 1, but slight for both in group 2 (**Table 4**).

#### Amount of information

Three out of four participants provided the same number of places where care was sought for diarrhoea (text message 13). Forty participants responded to text message 20 for which Fleiss-Cohen weighted kappa indicated non-significant (*P* = 0.38) slight agreement and Cicchetti-Allison borderline significant (*P* = 0.046) fair agreement (**Table 5**, Table S18 and S19 the **Online Supplementary Document(Online Supplementary Document)**).

**Table 5:** Amount of information, number of places recorded for total group (N = 409)

Text message: brief content	n of pairs	% agreement	Cicchetti-Allison and Fleiss-Cohen kappa	95% CI	*P*-value
13:where sought care for diarrhoea	4	3/4 agree*			
20:where sought care for fever or cough	40	75.0	0.23, 0.13	-0.06 to 0.53, -0.23 to 0.49	0.046†, 0.38

*Kappa cannot be calculated.

†*P* < 0.05.

### Reasons for differences in responses

Of the 226 participants in group 2, 51 participants provided 42 reasons for disagreement and 9 reasons were missing. For eight questions (text message 4, 6, 10a, 11, 12, 14, 15 and 16) there was disagreement between the face-to-face and text messaging answers. There were 17 reasons related to the text messaging method including 8 participants did not see the date (there were dates in the text messages to ask about the past two weeks), 3 misunderstood the question and 3 replied carelessly and did not pay attention. There were 22 reasons related to the face-to-face method including 8 participants did not know the accurate definition of a symptom, 7 misunderstood the question and 4 did not understand the date clearly. There were three other reasons that could not be directly related to the methods: two participants could not recall the information that was asked and one participant had a change of mind. (Table S20 in **Online Supplementary Document(Online Supplementary Document)**).

## DISCUSSION

### Principal results

This study showed that almost two-thirds of participants responded to the first text message about willingness to participate. Of the participants who responded that they were willing to participate, more than half completed the text messaging survey. A significantly higher proportion of participants not responding to the first question had children with a rural *Hukou* and received a lower median number of text messages. There were more children with diarrhoea in the non-completers group compared to the completers group, which may be explained by that caregivers of a child with diarrhoea had to answer the largest number of questions (at least 14 questions).A large number of text messages were manually sent of which only a few were in an incorrect format (researcher-related errors). Of the text message replies received, about a third were in an incorrect format (participant-related errors), but only a small proportion required follow-up. Overall, text message data were moderately to substantially equivalent to face-to-face data. The proportion of agreement was as follows for the 10 nominal dichotomous questions: >90% for 5 questions; between 80% and 90% for 4 questions; and 70.0% for 1 question. Kappa was as follows for these 10 nominal dichotomous questions: substantial for 6 (text message 4, 8, 14, 15, 16 and 18); fair for whether care was sought for diarrhoea (text message 11) and whether an ORS was given (text message 6); and could not be calculated for 2 questions (text message 5 and 7) that had an insufficient number of values for each cell in the 2x2 classification table. A calculation after the study finished showed that power was >0.95 for question 4, 14 and 15 which had kappa values of at least 0.70, while question 16 only had a power of approximately 0.60 with a kappa value of 0.65. However, we were unable to calculate agreement for the remaining questions because power was insufficient for these questions with small sample sizes. Finally, both the face-to-face and text message surveys generated errors in responses that caregivers gave.

### Strength and limitations

This is the first study using a cluster randomised crossover design to assess the validity of using text messaging for data collection in a middle-income country. Mobile phone use was high in our setting [53] and mobile phones were generally kept personally by the user. However, recruitment of caregivers was challenging and the text message response rate was insufficient for use in household surveys; these aspects were evaluated in another publication [50]. This allowed us to reach participants directly and ensure privacy. The findings of this study can be generalized to other rural areas in China with similar socio-economic characteristics, and levels of mobile phone use.

Despite stratified randomization, there were small differences in characteristics of the participants between group 1 and group 2. This may be explained by the cluster randomised design of the study in which it was more difficult to achieve balance between groups than in an individual randomised controlled study [57]. Participants in group 2 were less educated, lived more often in rural areas and were less likely to be the primary caregiver than participants in group 1, which may have contributed to a lower completion rate in group 2.

Four outcomes were part of six indicators for data quality [19,58]: data equivalence, amount of information, item response rate and completion rate. We could not assess two quality indicators: a validity check of the data against the true values and the absence of social desirability bias. We did not attempt to verify the reported diarrhoea or pneumonia symptoms because this was not the focus of our study.

Some of the kappa calculations were driven by low prevalence and we did not calculate a prevalence and bias adjusted kappa [59].

### Comparison with prior work

The previous text message data collection study in Zhao County found a lower initial response and completion rate, which may be explained by differences in study designs. However, both studies found substantial data agreement and similar reasons for differences [23]. Other studies that assessed data agreement mainly took place in high-income countries and also found a high degree of agreement [28,34-36,39,41,42].

Few grandparents could be included in the crossover study, because they were often unable to text message. This may be less of a problem in other settings where elderly people are more commonly able to text message [60]. Previous mHealth-based data collection studies frequently only included younger participants [28,29,32,33,35,37,61]. While in other settings socio-economic factors may influence usage of mobile phones [62], this did not seem to be of large influence in the study setting as the costs of text messaging were very low.

A study in the United Kingdom used an automated text messaging system, but out of 2952 text messages sent in total, still 214 (7%) text messages had to be sent manually [28]. As a result of system or researcher errors, about 6% of participants were sent the wrong number of text messages (too many or too few), while for about 2% of participants there were other problems [28]. However, an automated system would be a huge benefit for researchers. Sending text messages manually was a labour intensive process as one researcher (YL) was continuously sending text messages 12 hours a day (from 9 am till 9 pm) during the study period (14 days). Also before and after sending text messages, she had to do additional work for the study (communicate with the field workers and preparation and checking work). A private software company was used to provide support for the text messaging system. Open-source software is an important issue for mHealth-based research in low- and middle income countries, because limited budgets would benefit from free software. However, in some circumstances private software has value too [63].

### Future research

This study was an early attempt in a middle-income setting and thus more studies are required to address remaining questions. First, the response rate requires improvement as it was insufficient according to the requirements for a household survey (minimum response rate of 90%) [7]. There are many approaches that can increase the response rate which should be evaluated [13,23,50]. Text messaging could be a useful tool in remote rural areas, but our study showed that more rural participants refused to participate compared to people living in urban areas, which led to a lower response rate in rural areas. Second, while data equivalence is satisfactory for the questions that we were able to evaluate, there are still some questions with few responses where data equivalence has not yet been fully established. Further research is needed to establish the equivalence those questions. Third, cost-effectiveness is an important under-explored advantage of text messaging over current data collection methods and could reduce the need for resources, particularly in rural remote areas [13,22]. Fourth, operational guidelines need to be developed and tested to facilitate implementation of text messaging data collection [13].

## CONCLUSIONS

This study shows that text messaging produces data similar to data collected by face-to-face interviews in a middle-income setting, but several questions remain. Improving the response rate is important because text messaging surveys could be of greater value in rural remote areas due to the cost-saving potential [15].
