# Supplementary material for: Comparison of text messaging data collection vs face-to-face interviews for public health surveys: a cluster randomized crossover study of care-seeking for childhood pneumonia and diarrhoea in rural China
Source: J Glob Health. 2018 Jun 10;8(1):010802. doi: 10.7189/jogh.08.010802 (PMC5997366; doi:10.7189/jogh.08.010802)
Supplement: Online Supplementary Document [file jogh-08-010802-s001.pdf]

Online Supplementary Document

van Velthoven et al. Comparison of text messaging data collection vs face-to-face interviews for public health surveys: a cluster randomized crossover study of care-seeking for childhood pneumonia and diarrhoea in rural China

J Glob Health 2018;8:010802

Table S1 Descriptive information of text messaging data collection studies

First author year	Health purpose	Location	Data collection comparison	Study design	Participants
L'Engle 2012	Collect family planning information	Tanzania	No method comparison	Data collected during a 10-month pilot of the *Mobile for Reproductive Health programme*	2870 unique users of the programme, 56% male, 60% were aged 29 years or younger
Macedo 2012	Monitor patients with low back pain	Australia	No method comparison	Observational study nested within a randomised controlled trial over 12 months	105 trial participants, 41% male, aged 18-24 years
Suffolleto 2012 + Baird 2012 (comment paper)	Collect alcohol drinking data	United States	Within group: text messaging versus 28 day calendar-based recall method	-Randomised controlled trial with 3 groups: (i) intervention group with text message feedback; (ii) assessment group with no feedback; and (iii) control group (no alcohol-related text messages) -Data collection over 12 weeks	45 young adults identified as hazardous drinkers and owning mobile phone, 36% male, average of 21 years
Whitford 2012	Monitor infant feeding	Scotland, United Kingdom	Within group: Text message versus text	Data collection during a cohort study over 16	355 women from a cohort of recently delivered women, median age 29

			message, versus telephone call within 24 hours, versus same data collected from other sources and versus related measures	weeks	years
Moller 2012	Assess injury incidence in elite handball players	Denmark	No method comparison	Prospective cohort study over 31 weeks	342 elite handball players, 37% male, 38% aged younger than 16 years
Axen 2012	Monitor low back pain	Sweden	No method comparison	Prospective observational study over 6 months	262 patients with nonspecific low back pain, 52% male, median age 44 years
Magee 2011	Collect data during simulated disaster events	United States	No method comparison	Pilot project over 6 months	63 students, 25% male, median age 25 years
Schembre 2011	Track hunger ratings	Hawaii, United States	No method comparison	Feasibility study over 7 days	Convenience sample of 15 males (n=2) and females (n=13), age 21 years
Haberer 2010	Collect antiretroviral therapy adherence data	Uganda	Between groups:<br>-text messaging group<br>-interactive voice response group	-Randomised study<br>-Data collection over 3-4 weeks followed by qualitative interviews	19 trial participants (caregivers of human immunodeficiency virus-infected children), 10% male, median age 34 years
Johansen 2010	Monitor patients with low back pain	Denmark	Within group test-retest: text messaging versus retrospective telephone interview (recall past week, month and year)	Data collection over 53 weeks	25 patients with low back pain, mean age 41 years
Lim 2010	Collect sexual behaviour information	Australia	Between groups:<br>-text messaging group<br>-online diary group<br>-paper diary	Randomised controlled trial over 3 months	72 participants, 24 in each group, 28% male, median age 21 years

			group		
Alfven 2010	Monitor children with recurrent pain	Sweden	Within group test-retest:<br>-first verbal data collection in presence of physician<br>-second text messaging	-Test retest procedure with 3 day interval to measure reliability<br>-Response rate over 7 days<br>-Assessment of validity of scale	-Response rate: 15 children with recurrent pain, mean age 12 years<br>-Validity: 37<br>children, 16 boys, median age 13 years<br>-Reliability: 20 children, 11 boys, median age of 12 years
Kew 2010	Collect Irritable Bowel Syndrome symptom data	Malaysia	No method comparison	Cross sectional study conducted during a double blind randomised controlled trial, 10 weeks	38 undergraduates with Irritable Bowel Syndrome in a private medical university, 20 males, mean age 22
Kongsted 2009	Monitor patients with low back pain	Denmark	No method comparison	Longitudinal pilot study over 18 weeks	110 patients with low back pain, 50% male, mean age 43 years
Kuntsche 2009	Assess alcohol usage	Switzerlan d	Within group: Internet versus text messaging	Baseline Internet survey and text messaging data collection over 4 weekends	55 French speaking<br>individuals who answered all questions in the Internet<br>survey and participated in the text messaging survey for at least one entire weekend, 33% male, mean age 23 years
Roberts 2009	Audit day-case analgesia experiences	United Kingdom	Between groups:<br>-first audit postal survey<br>-second audit text messaging survey	Feasibility study, up to 2 days post-procedure	Patients who had<br>undergone day-case surgical procedures:<br>-62 in postal group<br>-25 text messaging group
Bexelius 2009	Influenza vaccination data collection	Sweden	Between groups:<br>-text messaging survey group<br>-telephone interview group	Feasibility study over approximately one week	-154 participants in text messaging group, 54% male, 60% aged 0-39 years<br>-1009 in telephone group<br>-Random sample of 4550 individuals aged 0–100 years, divided into two groups (2400 text messaging, 2150 telephone) was used

Haller 2009 +	Assess satisfaction with primary care consultation	Australia	Between groups:<br>-text messaging group<br>-card enquiry group	1:1 randomised controlled trial	402 consecutive patients:<br>-193 in text message group<br>-209 in card enquiry group
Haller 2006			No method comparison	Feasibility study	110 consecutive patients, 35% male, age 21 years
Anhøj 2004	Monitor patients with asthma	Denmark	No method comparison	Feasibility study over 2 months followed by focus group	12 participants, 6 males, median age 39 years, convenience sample of self-selected participants from a website

Table S2 Response rate, timeliness and data equivalence outcomes from text messaging data collection studies

Paper (first author, year)	Response rate	Timeliness	Data equivalence
L'Engle 2013	-Of 2870 people, 35% (n=995) reported gender, 32% (n=927) age, 29% (n=824) where they learned about the programme, 18% (n=509) an open-ended response<br><br>-Response rates to the open-ended question were similar across gender, age and promotion point categories<br><br>-67% of participants answered three or four questions and 33% answered only one or two questions	-	-
Macedo	-Response rates for text messaging alone ranged from 55-74%<br><br>-Text messaging supplemented with phone interviews ranged from 92-99%<br><br>- Participants completed a median number of 9 out of 12 assessments via text messaging (interquartile range, 5–11)<br><br>-No significant effect for any of the following predictors on response rate: age; sex; education level; pain levels at baseline; or pain improvement after 2 months' treatment	-	-
Suffolleto	-93% of participants in the	40% of replies	-Agreement between text message

2012+<br>Baird 2012 (comment paper)	assessment and intervention groups replied to the weekly text message drinking questions at least once<br>-73% of participants<br>in the assessment group and 80% in the intervention group completed all 12 weeks of queries	were sent within 1 minute in the assessment group compared with 65% in the intervention<br>group	and calendar: correlation 0.87 to 0.99 for maximum drinks and 0.73 to 0.97 for days drinking per week<br>-No differences in the proportion of subjects that would be categorised as either heavy drinkers or abstinent
Whitford 2012	-80% response rate (2372/2952 text messages)<br>-93% participant response rate (329/355 women)	-	-Reliability: kappa was 1.0 for a factual question and kappa was 0.80 for a numerical question<br>-Validity: kappa was 0.92 for text messaging data compared to a phone call within 24 hours and kappa was 0.85 for text messaging data compared with data from a health visitor<br>-Correlation validity was as expected for text responses compared to other demographic and clinical measures
Moller 2012	Weekly response rate ranged from 85% to 90% over 31 weeks	-	-
Axen 2012	Mean response rate for the text messages was 83% over 6 months, 90% in first week and 79% in last week<br>-93% participant response rate (244/262 participants)	-	-
Magee 2011	-Overall response rate of 77% (range 70-86%)<br>-94% of participants responded<br>-Significantly more likely to respond were: participants aged 24-29 (compared to those aged 18-23 years); graduates (compared to undergraduates); and participants with unlimited text message plans (compared to those without)	Median time to receive responses was 13.5 minutes and 80% of participants responded within 90 minutes	-
Schembre 2011	-Response rate was 75% (12/16 responses), 2 participants had <10 responses<br>-Minor variations by observation day or day of the week	93% of the ratings were received within 30 minutes	-
Haberer 2010	Text messaging weekly response rate 24% (0–33%) over 3-4 weeks		-
Johansen 2010	-	-	Test-retest reliability between text message and telephone interview: high proportion of agreement and small Bland-Altman average

			difference for the 1-week and 1-month recall, but very low proportion of agreement and high Bland-Altman average difference for 1-year recall
Lim 2010	-Proportion of incomplete diaries: 4% of text messaging diaries; 3% of paper diaries; and 1% of online diaries (*P*=0.001)<br>-90% (65/72) participants completed the end point: 23/24 from the text messaging group; 22/24 from the online group; and 20/24 from the paper group	Text messaging diaries were less likely to be submitted late than online diaries (*P*< 0.001)	-Kendall coefficients for numerical data: almost perfect agreement for one question; substantial agreement for three questions; and moderate agreement for one question (the diary collection mode did not affect the correlation)<br>-Kappa for binary categorical variables had substantial agreement on risk classification (kappa=0.74)
Alfven 2010	All children gave response to all three variables or to none	70% of responses to text messages were received within 15 and 83% within 60 minutes	-Construct validity between 2 scales measuring the same entity was 0.77<br>-Reliability test-retest: kappa for verbal scale was 0.73 and kappa for Visual Analogue Scale was 0.50
Kew 2010	-Response rate of 100% during 10 weeks<br>-33% were received on the following Monday without a reminder, 60% were received a day later after a single reminder, 6% 2-3 days later after 2-3 reminders and 2 non-responders were tracked down and met face-to-face	-	-
Kongsted	-101/110 patients responded to the first text message<br>-Follow-up rate declined as the study period went on with 86% in week 6, 78% in week 12 and 70% in week 18<br>-Drop-outs were more likely to be men, have presented to the chiropractor with acute low back pain and have leg pain in addition to low back pain	-	
Kuntsche 2009	Most participants (84%) answered all text message questions or left only one question unanswered	-	-Positive Bivariate Pearson correlation for usual quantity of alcohol between baseline Internet data and text messaging data<br>-Not always positive correlation for number of drinks indicated in the text messaging survey over the eight days of the survey
Roberts 2009	Completion rate was 69% (43/62) in	-	-

	the postal group and 40% (10/25) in the text messaging group which increased to 70% (a further 10) after sending a reminder		
Bexelius 2009	-40% (344 out of 868) responded to the first question<br><br>-15% (154 out of 1055 who had a listed mobile phone number) gave answers to all questions<br><br>-Text messaging gave 14 times higher nonparticipation rate compared with the telephone interview (odds ratio 14), partly explained by low extraction of mobile phone numbers from the telephone directory (44%)	-	No significant difference in data collected via text messaging and telephone interviews
Haller 2009	Response rate not significantly different between text message (80%) and card (86%)	-	Reported satisfaction not significantly different between text message (93%) and card (88%) when adjusted for clustering
Haller 2006	-	-	-
Anhøj 2004	-Half the participants replied to >2/3 of the requested data<br>-Median response rate was 69%<br>-Steady response rate, no signs of decreasing usage over time<br>-Out of a total of 727 study days, there were 423 days (58%) where participants replied to all questions, 31 days (4%) where they replied to some questions and 273 days (38%) where they did not reply at all	-	-

Table S3 Feasibility and acceptability to participants and researchers and cost outcomes from text messaging data collection studies

First author year	Feasibility and acceptability to participants	Feasibility and acceptability to researchers	Costs
L'Engle 2013	Adolescents and young adults were the heaviest users of the programme among those reporting their age	-	-
Macedo 2012	-Of 133 participants of a larger trial, 105 (61%) had a mobile phone and 97 (56%) knew how to use text messages and were included<br><br>-12 out of 38 (32%) people older than 60 years owned and used a mobile phone for text messaging	-	-

Suffolleto 2012 Comment	One participant was excluded because of not owning a mobile phone	-	-
Whitford 2012	The text messaging survey was found to be convenient and acceptable	-Text messaging was a functional and easy method of gathering a large volume of data<br>-Text messages were sent as scheduled to 92% of participants; however, because of system or researcher errors, 6% were sent the wrong number of texts, 2% had other problems	-
Moller 2012	-	-	-
Axen 2012	-Text messaging method was found to be user friendly<br>-Various reasons for drop-out: no explanation (5); impossible to reach by telephone and mail (7); two had neck pain as their primary complaint (2); thought it was silly to answer ‘‘0’’ all the time (1); found the text messages too costly (1); did not have the time to answer (1); or could not remember the degree of bothersomeness (1)	-	-
Magee 2011	-73% agreed or strongly agreed that they were happy to participate<br>-82% said they would do the study again<br>-10% agreed that the study was a waste of time 71% recommended the study to their friends<br>-47% agreed that they should be compensated for their participation	-	-
Schembre 2011	-	Of 168 text messages, 3% were undelivered because of mobile service interruptions	-
Haberer 2010	-31 out of 121 participants of a larger trial owned a mobile phone, 10 either no longer had their own phones or had non-functional phones at the time the study began, one participant had a landline telephone<br>-Poor understanding how to respond to the interactive voice call and text messaging prompts<br>-Lack of understanding during training sessions	-	-Initial set up costs US $1,900 for interactive voice call programming, US$1,000 for text messaging programming and US$300 to establish both systems with local mobile phone network<br>-Ongoing costs US$113

	-Despite problems, technologies were acceptable		for airtime (US$0.23 per minute and US$0.05 per text message) during the 4-week period
Johansen 2010	All 25 contactable participants found that a third text message question would have been acceptable	-	-Total costs for text messaging was €9530 (€8700 system lease<br><br>+ €830 for sending text messages)<br><br>-Text messaging was considerably less costly than a paper-based survey, beyond a threshold number of questionnaires
Lim 2010	-All but one participant (online group) completed at least one diary<br><br>-51% preferred online, 38% preferred text messaging and 8% preferred paper<br>-Similar proportions of participants within the text messaging group (70%) and online group (73%) preferred their assigned method<br>-Text messaging participants were more uncertain about how to complete the diary than those in the online group (*P*=0.047)	-	-
Alfven 2010	-All children had a mobile phone of their own<br><br>-Children found it easy to describe in figures on text messages their pain intensity, pain duration and pain-related disability	-	Once programmes are built, the costs are low because of use of participants' mobile phones and low costs of text messages
Kew 2010	-Participants were given the choice between email and text messaging and all chose text messaging<br>-No one reported difficulty using the simple codes to submit symptom reports	-	-
Kongsted	-	No manual entry of data meant fewer human resources and avoiding potential errors	-
Kuntsche 2009	-	-	-
Roberts 2009	-	-	-

Bexelius 2009	-	-	-
Haller 2009	-Out of 450 young people 409 (91%) had a mobile phone, 7 (1.6%) had to be excluded because they did not want to provide their mobile phone number for research purposes<br><br>-Several participants sent support and best wishes messages for the trial together with their texted response, none sent negative or unpleasant messages, no comments were found on any of the response cards	-	-
Haller 2006	-91% (96/110) agreed to participate<br>-91% (87/96) had a mobile phone and 85/87 agreed to provide a phone number for research<br>-No one expressed concern about paying for text message replies	-Could not send text messages near medical equipment because of safety concerns<br>-Inadequate mobile network coverage in the rural practice led to delays in sending messages	Cost of sending a text message (approximately US$0.25) slightly exceeded the usual cost of printed material
Anhøj 2004	-Participants were enthusiastic about the text message diary; it became an integrated part of everyday life<br>-Participants wished for a simpler diary with only one text message to respond to and a system with a Web interface for system customization and graphical display of diary data history<br>-The self-reported prior experience with text messages was moderate for the majority of the participants; one participant had never used text messaging prior to this study, five were medium users receiving and sending 1 to 3 messages daily, three were heavy users receiving and sending more than 4 messages daily<br>-None of the participants had used a questionnaire based on text messaging prior to the study<br>- For one participant, not having used text messaging before the study was not an obstacle to participation	-	-

Table S4 Final face-to-face and text message questionnaires used in study

Face-to-face questions		Text message questions	
**Identification number of question and content in English**	**Content in Chinese characters**	**Text message number and content in English**	**Content in Chinese characters**
NA		1.Hello, this is Zhao County's Maternal and Child Health Hospital. We want to ask you questions about your youngest child's health. Your responses to our questions are meaningful to us, it can help us improving child health. [*Zhao County's Maternal and Child Health Hospital message*]	您好，我们是赵县妇幼，想了解您家里最小的那个孩子健康有关情况。您的回复对我们很重要，可以帮助我们改善儿童健康状况。【县妇幼短信】
NA		2.You do not have to pay extra fees and you will be paid back ¥0.1 for replying to messages. In addition, if you answer all the questions, you will receive ¥5 extra recharged on your mobile phone credit within 2 weeks. Are you willing to answer questions?<br>Please respond: I am willing/I am not willing [*Zhao County's Maternal and Child Health Hospital message*]	回短信无额外费用，并会返还短信费 1 角/条。答完所有问题可另得 5 元话费。2 周内充到您手机。您愿意回答吗？<br>请回复：愿意/不愿意【县妇幼短信】
NA		3.How does your child call you (the relationship between you and your child)?<br>Please respond: mother/father/grandmother/grandfather/other…..(please specify) [*Zhao County's Maternal and Child Health Hospital message*]	您的孩子管您叫什么（您与孩子的关系）？请回复：妈妈/爸爸/奶奶/爷爷/其他（请列出）【县妇幼短信】

DI.1. Has (*name)*[*] had diarrhoea in the last 2 weeks (please think back from two weeks ago till today? Diarrhoea is the passage of 3 or more loose or watery stools, compared to usual, per day.<br>1.Yes<br>2.No →***Skip to CO.1***<br>8.Do not know→***Skip to CO.1***	孩子过去两星期内（从今天算起往前推 2 星期，并强调日期）腹泻（拉稀/拉肚子）了吗？<br>腹泻定义为一天稀便(和平常相比)或水样便三次或以上。<br>1.是<br>2.否 ——>结束 DI 部分，转到 CO.1<br>8.不知道 ——>结束 DI 部分，转到 CO.1	4.Diarrhoea is the passage of 3 or more loose or watery stools, compared to usual, per day. Has your youngest child had diarrhoea in the last 2 weeks (from *month/day* till *today*)? Please respond: child had diarrhoea/child didn't have diarrhoea [*Zhao County's Maternal and Child Health Hospital message*]<br>***When child didn't have diarrhoea→skip to text message 14***	拉肚子（拉稀）指比平常稀的大便或水样便一天三次或以上。您家最小孩子过去两周内（…月…日到…今天）拉肚子/拉稀了吗？<br>请回：拉了/没拉【县妇幼短信】
DI.2.Did (*name*) have blood in the stools?<br>1.Yes<br>2.No<br>8.Do not know	孩子大便中带血吗？(由于腹泻导致的大便带血)<br>1.是<br>2.否<br>8.不知道	5.Did your youngest child have blood in the stools (caused by diarrhoea)? Please respond: child had blood in stools/child did not have blood in stools [*Zhao County's Maternal and Child Health Hospital message*]	您家最小的孩子有过因拉肚子/拉稀导致的大便带血（拉粑粑里边带血）吗？<br>请回复：带血/不带血【县妇幼短信】
DI.3.During this last episode of diarrhoea, did (*name*) drink any of the following: *read each item aloud and record response before proceeding to the next.*<br>A fluid made from a packet called ORS?<br>1.Yes<br>2.No<br>8.Do not know	孩子在最近一次腹泻期间，你是否给了以下液体：（逐项读出，并记录每一个选项及答案。）<br>1.口服补液盐（一种治疗拉稀的药物）<br>1.是<br>2.否<br>8.不知道	6.During this last episode of diarrhoea, did your youngest child drink a fluid made from a packet called ORS (drug for diarrhoea treatment)? Please respond: child had ORS/child did not have ORS [*Zhao County's Maternal and Child Health Hospital message*]	您最小孩子最近一次拉肚子/拉稀期间，喝了口服补液盐吗？(一种治疗拉稀的药)请回复：喝了口服补液盐/没喝口服补液盐【县妇幼短信】
One of the following fluids: breast milk, formula, tap water, mineral water, rice water or soup?	2.母乳、配方奶、白开水、矿泉水、米汤、菜汤<br>1.是	7.During this last episode of diarrhoea, did your youngest child drink one of the following fluids: breast milk, formula, tap	您最小孩子最近拉肚子/拉稀期间，是否喝了以下液体：母乳、配方奶、白开水、矿泉水、米汤、

1.Yes<br>2.No<br>8.Do not know	2.否<br><br>8.不知道	water, mineral water, rice water or soup?<br>Please respond: child drank one or more of those fluids/child did not drink those fluids [*Zhao County's Maternal and Child Health Hospital message*]	菜汤？<br><br>请回：喝过这些/都没喝过【县妇幼短信】
Other fluids such as tea, drinks, water with honey or any sugary drinks?<br>1.Yes<br>2.No<br>8.Do not know	3. 其他液体， 如茶或饮料、蜂蜜、甜水等<br><br>1.是<br><br>2.否<br><br>8.不知道	8.During this last episode of diarrhoea, did your youngest child drink other fluids such as tea, drinks, water with honey or any sugary drinks?<br>Please respond: child drunk other fluids/child did not drink other fluids [*Zhao County's Maternal and Child Health Hospital message*]	您家最小的孩子最近一次拉肚子/拉稀期间，是否喝了其他液体， 如茶、饮料、蜂蜜水或任何甜水等？<br><br>请回复：喝过其他液体/没喝其他液体【县妇幼短信】
DI.3.a During (*name's*) diarrhoea, did he/she drink much less, about the same, more than usual or none? *If less, probe*: Was he/she offered much less than usual to drink or somewhat less?<br>1.None<br>2.Much less<br>3.Somewhat less<br>4.About the same<br>5.More<br>8.Do not know	孩子最近一次腹泻时，孩子喝的汤水和母乳是比平常少、与平常一样多、比平常多还是什么都没喝？（包括任何孩子能喝的东西， 如母乳和配方奶） [如果比平常少， 继续问：比平常少很多， 还是少一点？]<br><br>1.什么也没喝<br><br>2.少得多<br><br>3.少一点<br><br>4.一样多<br><br>5.比平常多<br><br>8.不知道	9.During your youngest child's diarrhoea, how much did he/she drink compared to usual (anything the child can drink, including breast milk or formula)?<br>Please respond: none/much less /somewhat less/about the same/more [*Zhao County's Maternal and Child Health Hospital message*]	您最小孩子那次拉肚子期间， 喝的（任何能喝的， 包括母乳和配方奶）比平常怎样？<br><br>请回：一点没喝/少得多/少些/一样/多些【县妇幼短信】

DI.3.b When (*name*) had diarrhoea, did he/she eat less, about the same, more food than usual or none?<br>*If less, probe*: Much less or a little less?<br>1.None<br>2.Much less<br>3.Somewhat less<br>4.About the same<br>5.More<br>6.Child never received solid or semi-solid foods<br>8.Do not know	孩子最近一次腹泻期间，吃的物件是比平常少、与平常一样多、比平常多还是什么都没吃？[如果比平常少，继续问：比平常少很多，还是少一点？如果没有吃，继续问：是从没吃过还是仅腹泻期间没有吃]<br>1.什么也没吃<br>2.少很多<br>3.少一点<br>4.一样多<br>5.比平常多<br>6.孩子还没有吃过任何固体或半固体的食物<br>8.不知道	10.Has your youngest child ever been introduced to foods such as rice, noodles, manto, meat, eggs, vegetables, fruits (excluding breast milk or formula)?<br>Please respond: child received foods before/child never received foods before [*Zhao County's Maternal and Child Health Hospital message*]<br>***When child never received foods before→skip to text message 11***<br>10a.When your youngest child had diarrhoea, how much did he/she eat (including all foods, excluding breast milk and formula) compared to usual?<br>Please respond: none/much less/somewhat less/about the same/more [*Zhao County's Maternal and Child Health Hospital message*]	您最小的孩子吃过米饭、面条、馒头、肉、蛋、蔬菜或水果这样的物件吗？（除母乳及配方奶）<br>请回：吃过物件/从没吃过物件【县妇幼短信】<br>对已经添加了辅食的孩子发送：<br>您最小孩子那次拉肚子期间，吃物件（母乳和配方奶不算）的量比平常怎样？<br>请回：没吃/少很多/少些/一样/多些？【县妇幼短信】
DI.4During this last episode of diarrhoea in (*name*), did you seek advice or treatment for the diarrhoea outside the home<br>1.Yes, outside the home→*skip to DI.4.b*<br>2.No, managed at home<br>8.Do not know	孩子最近这次腹泻期间，除了住在一起的家人，您还寻求过指导或治疗吗？<br>1.是，到家庭外寻求指导或治疗 ——>转到DI.4.b<br>（包括询问邻居、去医院、为孩子举行宗教仪式等。如医生到家中给予指导或治疗，也算在内。当母亲外出寻求指导或治疗时，是否带孩子一起去没有关系，如	11.During this last episode of diarrhoea in your youngest child, did you seek advice or treatment outside the home (ask anyone who is not a family member living with you)?<br>Please respond: yes, outside the home/no, at home [*Zhao County's Maternal and Child Health Hospital message*]<br>***When response is "yes outside the home" skip to text message 13***	您最小孩子最近这次拉肚子/拉稀时，除了住在一起的家人，您还寻求过指导或治疗了吗？<br>请回复：寻求过/没有寻求【县妇幼短信】

	去药店买药没带孩子，也算在内；不要读出）<br><br>2. 否，在家里自行处理——>转到 DI.4a<br><br>8. 不知道 ——>结束本部分，转到下一部分		
DI.4.a During this last episode of diarrhoea in (*name*), why didn't you seek advice? *Only one answer allowed.*<br>01.Mild disease/did not need outside help<br>02.Geographical access (too far from facility)<br>03.Costs (had to pay for visit or transportation)<br>04.Facility closed/staff not available<br>05.Poor quality of care at facility<br>06.Not necessary<br>07.Religious beliefs<br>08.Other: specify<br>________<br>88.Do not know	孩子最近这次腹泻期间，没有寻求指导或治疗的主要原因？——> 答完此题，结束本部分<br><br>01.不严重/不需要别人帮助<br><br>02.家离卫生机构太远<br><br>03.费用（没钱支付服务或交通费）<br><br>04.卫生机构没有人/没有开门<br><br>05.卫生机构的服务质量差<br><br>06.没必要<br><br>07.宗教信仰<br><br>08.其他：_______<br><br>88.不知道	12.During this last episode of diarrhoea in your youngest child, why did you not seek advice diarrhoea outside the home?<br>Please respond by giving us one reason, the most important one. [*Zhao County's Maternal and Child Health Hospital message*]	您最小孩子最近这次拉肚子/拉稀时，除了住在一起的家人，您为什么没有寻求指导或治疗?<br><br>请回复一个最主要原因【县妇幼短信】
DI.4.b Where did you seek care when (*name*) had diarrhoea?<br>*Record all sources mentioned. Prompt: "Anywhere else?"*<br><u>1.Relative or friend</u><br>11.Own family<br>1.Yes 2.No<br>12.Friends or neighbours<br>1.Yes 2.No<br><u>2.Health facility</u><br>21.County level hospital	孩子最近这次腹泻期间，你到哪里寻求指导和治疗？（不要念出选项，记录所有的提到的地方。可提示"还有其他地方吗？"）<br><br>1.家中亲友	13.During this last episode of diarrhoea in your youngest child, where did you seek advice or treatment when your youngest child had diarrhoea?<br>Please respond by telling us all the places you went to. [*Zhao County's Maternal and Child Health Hospital message*]	您最小孩子最近那次拉肚子/拉稀时您到哪里寻求指导或治疗？请回复去的所有地方。【县妇幼短信】

or above (excluding MCH hospital)<br>1.Yes 2.No<br>22.County level MCH hospital<br>1.Yes 2.No<br>23.Community health centre<br>1.Yes 2.No<br>24.Township hospital<br>1.Yes 2.No<br>25.Community health station<br>1.Yes 2.No<br>26.Village clinic<br>1.Yes 2.No<br><u>3.Private health facility</u><br>31.Private hospital<br>1.Yes 2.No<br>32.Private clinic<br>1. Yes. 2.No<br>33.Pharmacy<br>1.Yes 2.No<br><u>4.Community</u><br>41.Midwife<br>1.Yes 2.No<br>42.Staff for family planning<br>1.Yes 2.No<br><u>5.Other: </u>1.Yes 2.No<br>Specify: __	11 自己的家人<br>1.是 2.否<br>12 朋友或邻居<br>1.是 2.否<br>2.公立医疗机构<br>21.县级及以上医院（不含妇幼保健院）<br>1.是 2.否<br>22 县级及以上妇幼保健院<br>1.是 2.否<br>23.社区卫生中心<br>1.是 2.否<br>24.乡镇卫生院<br>1.是 2.否<br>25.社区卫生站<br>1.是 2.否<br>26.村卫生室<br>1.是 2.否<br>3.私营医疗机构<br>31.私营医院<br>1.是 2.否<br>32.个体诊所<br>1.是 2.否<br>33.药店/药贩<br>1.是 2.否	***After response to 13 prompt:***<br>13a.Did you go anywhere else during the last episode of diarrhoea in your youngest child? Please respond by telling us all the places you went to. [*Zhao County's Maternal and Child Health Hospital message*]	回复后，追问：<br>最小孩子最近那次拉肚子/拉稀时，您还去过其他地方吗？请回复列出所有去的地方。【县妇幼短信】

	4. 社区<br>41.接生员<br>1.是 2.否<br>42.计生干事<br>1.是 2.否 5.其他：1.是 2.否____		
CO.1.Has (*name*) been ill with a fever at any time in the last 2 weeks, (please think back from two weeks ago till today)?<br>1.Yes<br>2.No<br>8.Do not know	您最小孩子在过去 2 星期内（从今天算起往前推 2 星期，并强调日期）发过烧吗？<br>1.是<br>2.否<br>8.不知道	14.Has your youngest child had fever at any time in the last 2 weeks (from *month/day* till *today*)?<br>Please respond: child had fever/child did not have fever [*Zhao County's Maternal and Child Health Hospital message*]	您最小孩子在过去 2 周内（…月…日到…今天）有过发烧吗？<br>请回复：发烧了/没发烧【县妇幼短信】
CO.2.Has (*name*) had an illness with a cough at any time in the last 2 weeks (please think back from two weeks ago till today)?<br>1.Yes<br>2.No<br>8.Do not know	您最小孩子在过去 2 星期内（从今天算起往前推 2 星期，并强调日期）生过病，且生病时有过咳嗽吗？<br>1.是<br>2.否<br>8.不知道	15.Has your youngest child had cough caused by illness at any time in the last 2 weeks (from *month/day* till *today*)?<br>Please respond: child had cough caused by illness/child did not have cough caused by illness [*Zhao County's Maternal and Child Health Hospital message*]	您最小孩子在过去 2 周内（…月…日到…今天）有过因为生病引起咳嗽吗？<br>请回复：有过生病引起的咳嗽/没有过生病引起的咳嗽【县妇幼短信】
CO.2.a.Did (*name*) breathe faster than usual with short, fast breaths or have difficulty breathing *(local terms)* in the last 2 weeks (please think back from two weeks ago till today)?<br>1.Yes<br>2.No→***skip to* CO.3**<br>8.Do not know→ ***skip to* CO.3**	您最小孩子过去 2 星期内（从今天算起往前推 2 星期，并强调日期），呼吸是否比平时快而短，或有呼吸困难（喘不上气/憋得慌）吗？<br>1.是<br>2.否——>转到 CO.3 核查 8.不知道——>转到 CO.3 核查	16.Did your youngest child breathe faster than usual with short, fast breaths or have difficulty breathing in the last 2 weeks (from *month/day* till *today*)?<br>Please respond: child had/child didn't have [*Zhao County's Maternal and Child Health Hospital message*]<br>***When response is "no" go to "check answers"***	您最小孩子 2 周内（…月…日到…今天）有过呼吸比平时快而短，或者有喘不上气/憋得慌吗？<br>请回复：有过/没有【县妇幼短信】

CO.2.b Were the symptoms due to a problem in the chest or a blocked nose?<br>1.Problem in the chest→ ***skip to* CO.3**<br>2.Blocked nose<br>3.Both→ ***skip to* CO.3**<br>4.Other, specify: ____→ ***skip to* CO.3**<br>8.Do not know→ ***skip to* CO.3**	这些症状是因为肺部有问题还是因鼻塞引起的？<br><br>1.肺部问题——>转到 CO.3 核查<br><br>2.鼻腔堵塞——>转到 CO.3 核查<br><br>3.两者都有——>转到 CO.3 核查<br><br>4.其他原因______——>转到 CO.3 核查<br><br>8.不知道——>转到 CO.3 核查	17.What's the reason for the fast breathing or difficult breathing? Please respond: problem in the chest/blocked nose/both problem in the chest and blocked nose/other reason……(please give reason) [*Zhao County's Maternal and Child Health Hospital message*]	您的孩子呼吸快或喘不上气/憋得慌是因为什么？<br><br>请回复：肺部问题/鼻腔堵塞/两者都有/其他原因（请列出）【县妇幼短信】
**CO.3**<br>***Check answers in CO.1 for fever and CO.2 for cough:***<br><br>***"No" for fever and "No" for cough→skip to end***<br><br>***"Yes" for and/or "Yes" for cough →ask CO.4***	调查员核查：<br><br>CO.1 或<br><br>CO.2<br><br>1.只要有一项选"1.是"——> 继续问 CO.4 治疗指导；<br><br>2.都没有选"1.是"——>结束 CO 部分，转到下一部分	***Check answers in text message 14 for fever and 15 for cough.***<br><br>***"No" for fever and "No" for cough→sent text message 21***<br><br>***"Yes" for fever and/or "Yes" for cough→ send text message 18***	
CO.4During this last episode of fever or cough in (*name*), did you seek advice or treatment for the fever/cough outside the home?<br>1.Yes→***skip to* CO. 4.b**<br>2.No→***skip to* CO.4.a**<br>8.Do not know	您最小孩子最近这次发烧/咳嗽时，除了住在一起的家人，您还寻求过指导或治疗吗？<br><br>1.是，到家庭外寻求指导或治疗——>转到 CO.4.b<br><br>（"到家庭以外寻求指导或治疗"包括询问邻居、去医院、为孩子举行宗教仪式等。医生到	18.During this last episode of fever or cough in your youngest child, did you seek advice or treatment outside the home (ask anyone who is not a family member living with you)? Please respond: yes, outside the home/no, at home. [*Zhao County's Maternal and Child Health Hospital message*]	您最小孩子最近那次发烧/咳嗽时，除了住在一起的家人，您还寻求指导或治疗了吗？<br><br>请回复：寻求过/没有寻求【县妇幼短信】

	家中给予指导或治疗，也算在内。当母亲外出寻求指导或治疗时， 是否带孩子一起去没有关系， 如去药店买药没带孩子， 也算在内。不要读出）<br><br>2.否， 在家自行处理——>转到 CO.4.a<br><br>8.不知道——>结束本部分， 转到下一部分		
CO.4.a During this last episode of fever or cough in (*name*), why didn't you seek advice? Only one answer allowed.<br>01.Mild disease/did not need outside help<br>02.Geographical access (too far from facility)<br>03.Costs (had to pay for visit or transportation)<br>04.Facility closed/staff not available<br>05.Poor quality of care at facility<br>06.Not necessary<br>07.Religious beliefs<br>08.Other: specify<br>________<br>88.Do not know	您最小孩子最近这次发烧/咳嗽时， 没有寻求指导或治疗的主要原因？ [单选] ——> 答完此题， 转到下一部分<br><br>01.不严重/不需要别人帮助<br><br>02.家离卫生机构太远<br><br>03.费用（没钱支付服务或交通费）<br><br>04.卫生机构没有人/没有开门<br><br>05.卫生机构的服务质量差<br><br>06.没必要<br><br>07.宗教信仰<br><br>08.其他：_______<br><br>88.不知道	19.During this last episode of fever or cough in your youngest child, why did you not seek advice outside the home?<br>Please respond by giving us one reason, the most important one. [*Zhao County's Maternal and Child Health Hospital message*]	您最小孩子最近那次发烧/咳嗽时除了住在一起的家人， 您为什么没有寻求指导或治疗?<br><br>请回复一个最主要原因【县妇幼短信】
CO.4.b Where did you seek advice or treatment when (*name*) had fever or cough? *Record all sources mentioned. Prompt:*	您最小孩子最近那次发烧/咳嗽时， 到哪里寻求指导或治疗？[记录	20.During this last episode of fever or cough in your youngest child, where did you seek advice or treatment	您最小孩子最近那次发烧/咳嗽时您到哪里寻求指导或治疗？

"Anywhere else?"<br>1.Relative or friend<br>11.Own family<br>1.Yes 2.No<br>12.Friends or neighbours<br>1.Yes 2.No<br>2.Health facility<br>21.County level hospital or above (excluding MCH hospital)<br>1.Yes 2.No<br>22.County level MCH hospital<br>1.Yes 2.No<br>23.Community health centre<br>1.Yes 2.No<br>24.Township hospital<br>1.Yes 2.No<br>25.Community health station<br>1.Yes 2.No<br>26.Village clinic<br>1.Yes 2.No<br>3.Private health facility<br>31.Private hospital<br>1.Yes 2.No<br>32.Private clinic<br>1.Yes. 2.No<br>33.Pharmacy<br>1.Yes 2.No<br>4.Community<br>41.Midwife<br>1.Yes 2.No<br>42.Staff for family planning<br>1.Yes 2.No<br>5.Other: 1.Yes 2.No<br>Specify: _	提到的所有地点。可提示"还有其他地方吗？"]<br><br>1.家中亲友<br><br>11 自己的家人<br><br>1.是 2.否<br><br>12 朋友或邻居<br><br>1.是 2.否<br><br>2.公立医疗机构<br><br>21.县级及以上医院（不含妇幼保健院）<br><br>1.是 2.否<br><br>22.县级及以上妇幼保健院<br><br>1.是 2.否<br><br>23.社区卫生中心<br><br>1.是 2.否<br><br>24.乡镇卫生院<br><br>1.是 2.否<br><br>25 社区卫生站<br><br>1.是 2.否<br><br>26.村卫生室<br><br>1.是 2.否<br><br>3.私营医疗机构<br><br>31.私营医院<br><br>1.是 2.否<br><br>32.个体诊所	when your youngest child had fever or cough? Please respond by telling us all the places you went to. [*Zhao County's Maternal and Child Health Hospital message*]<br><br>***After response to 20 prompt:***<br>20a. Did you go anywhere else during the last episode of fever and cough in your youngest child? Please respond by telling us all the places you went to. [*Zhao County's Maternal and Child Health Hospital message*]	请回复列出去的所有地方。【县妇幼短信】<br><br>回复后，追问：<br><br>最小孩子最近那次发烧/咳嗽时，您还去过其他地方吗？请回复列出所有去的地方。【县妇幼短信】

	1.是 2.否<br>33.药店/药贩<br>1.是 2.否<br>4. 社区<br>41.接生员<br>1.是 2.否<br>42.计生干事<br>1.是 2.否<br>5.其他：1.是 2.否<br>________		
NA		21.This is the end of the survey. Thank you very much for participating! You will receive ¥0.1 per text message and ¥5 for participating automatically on your mobile phone credit within two weeks. [*Zhao County's Maternal and Child Health Hospital message*]	调查结束，非常感谢您的参与！您将在 2 星期内得到返还的短信费及 5 元话费的补偿，将直接充到您的手机上。【县妇幼短信】

Table S5. Number of cross-over study participants recruited in villages per group (N=1014)

Number[a]	Group 1 (n=371)			Group 2 (n=643)		
	Village number	Number of participants	%	Village number	Number of participants	%
1	1	15	4.0	2	17	2.6
2	3	13	3.5	5	7	1.1
3	4	21	5.7	7	11	1.7
4	10	18	4.9	8	24	3.7
5	12	15	4.0	9	23	3.6
6	13	43	11.6	11	16	2.5
7	16	15	4.0	15	7	1.1
8	17	27	7.3	18	12	1.9
9	20	47	12.7	19	14	2.2
10	27	4	1.1	21	24	3.7
11	32	41	11.1	22	10	1.6
12	36	33	8.9	23	15	2.3
13	40	31	8.4	24	24	3.7
14	43	37	10.0	25	4	0.6
15	46	11	2.8	26	12	1.9
16				28	16	2.5
17				29	36	5.6
18				31	34	5.3
19				33	38	5.9
20				34	31	4.8
21				35	43	6.7
22				37	47	7.3
23				38	15	2.3
24				39	19	3.0
25				41	38	5.9
26				42	89	13.9
27				44	17	2.4

[a]In group 1, the total number of villages was 15 with a median number of 20 participants (Q1-Q3; 13-36) per village. In group 2, the total number of villages was 27 with a median number of 33 participants (Q1-Q3; 22-39) per village.

Table S6. Characteristics of cross-over study participants

Variables	Total (N=1014)	Group 1 (n=371)	Group 2 (n=643)	Comparison Statistics[a]	P value
**Use smartphone, n (%)**				χ^2=0.005	0.95
Yes	455 (44.9)	167 (45.0)	288 (44.8)		
No	559 (55.1)	204 (55.0)	355 (55.2)		
**Primary usage mobile phone for calls and text messages, n (%)**				Fisher's exact test	0.96
Calling	811 (80.0)	297 (80.0)	514 (79.9)		
Text messaging	29 (2.9)	10 (2.7)	19 (3.0)		
Both in equal measure	170 (16.8)	62 (16.7)	108 (16.8)		
Other[b]	4 (0.3)	2 (0.6)	2 (0.3)		
**Primary usage mobile phone for *QQ* and text messages, n (%)**				Fisher's exact test	0.21
Text message	330 (32.5)	124 (33.4)	206 (32.0)		
QQ	385 (38.0)	147 (39.6)	238 (37.0)		
Both in equal measure	86 (8.5)	22 (5.9)	64 (10.0)		
Do not use either	211 (20.8)	77 (20.8)	134 (20.8)		
Other (seldom use them (2))	2 (0.2)	1 (0.3)	1 (0.2)		
**Number of calls made per week, median (Q1-Q3)**	10 (7-20)	10 (7-21)	10 (7-20)	MWU/WW z=-1.29	0.20
Do not know number of calls made per week, n (%)	156 (15.3)	47 (12.4)	109 (17.1)		
**Number of calls received per week, median (Q1-Q3)**	10 (7-20)	10 (6-21)	10 (7-20)	MWU/WW z=-1.11	0.27
Do not know number of calls received per week, n (%)	142 (14.0)	48 (12.7)	94 (14.7)		
**Number of text messages sent per week, median (Q1-Q3)**	1 (0-7)	1 (0-7)	2 (0-8)	MWU/WW z=-0.68	0.49
Do not know number of text messages sent per week, n (%)	59 (5.8)	22 (5.9)	37 (5.9)		
**Number of text messages received per week, median (Q1-Q3)**	10 (5-15)	8 (5-15)	10 (5-15)	MWU/WW z=-0.76	0.45
Do not know number of text messages received per week, n (%)	80 (8.0)	26 (6.9)	54 (8.5)		
**Households having phone number of any health facility, n (%)**				Fisher's exact test	0.06
Yes	662 (65.3)	245 (66.0)	417 (64.9)		
No	343 (33.8)	126 (34.0)	217 (33.8)		
Do not know	9 (0.9)	0 (0.0)	9 (1.3)		

**Households having phone number of county hospital or above, n (%)**[d]				χ^2= 4.12	0.13
Yes	296 (29.2)	102 (27.5)	194 (30.2)		
No	704 (69.4)	267 (72.0)	437 (68.0)		
Do not know	14 (1.4)	2 (0.5)	12 (1.8)		
**Households having phone number of township hospital, n (%)**				χ^2= 10.78	0.005[c]
Yes	227 (22.4)	101 (27.2)	126 (19.6)		
No	772 (76.1)	268 (72.2)	504 (78.4)		
Do not know	15 (1.5)	2 (0.6)	13 (2.0)		
**Households having phone number of village clinic, n (%)**				Fisher's exact test	0.09
Yes	496 (48.9)	182 (49.1)	314 (48.8)		
No	510 (50.3)	189 (50.9)	321 (49.9)		
Do not know	8 (0.8)	0 (0.0)	8 (1.3)		
**Number of times mobile phone was used to obtain health information in past 3 months, n (%)**				MWU/WW z=-1.54	0.12
Never	646 (63.7)	248 (66.8)	398 (61.9)		
Once	50 (4.9)	17 (4.6)	33 (5.1)		
Twice	64 (6.3)	19 (5.1)	45 (7.0)		
Three times	39 (3.8)	16 (4.3)	23 (3.6)		
More than three times	215 (21.3)	71 (19.2)	144 (22.4)		
**Would like to use mobile to receive health information, n (%)**				χ^2= 2.56	0.26
Yes	603 (93.3)	236 (95.2)	367 (92.2)		
No	25 (3.9)	8 (3.2)	17 (4.3)		
Other (neutral (1), it is hard to tell (1), it does not matter to have it or not (16))	18 (2.8)	4 (1.6)	14 (3.5)		

[a]Chi-square (χ^2), Mann-Whitney U/Wilcoxon W (MWU/WW), quartile (Q), z-score (z).
[b]QQ (1), depends (1), neither (2).
[c]P<0.05
[d]including county hospital, county children's hospital, private hospital.

Table S7. Item response rate and overall response rate

Text message question number: brief content	Total group (N=1014)			Group 1 (n=371)			Group 2 (n=643)			Comparison	
	n respond	n text messages sent	Response rate (%)	n respond	n text messages sent	Response rate (%)	n respond	n text messages sent	Response rate (%)	X^2	*P* value
2: responded to willingness to participate (including those who responded 'no', meaning not willing)	662	1014	65.3	233	371	62.8	429	643	66.7	1.59	0.21
2: said "yes" for willingness to participate	651	1014	64.2	229	371	61.7	422	643	65.6	1.56	0.21
3: which caregiver	585	651	89.9	203	229	88.6	382	422	90.5	0.57	0.45
4: child had diarrhoea	538	585	92.0	189	203	93.1	349	382	91.4	0.54	0.46
5: child had blood in stools	41	44	93.2	14	14	100.0	27	30	90.0	-	0.54[a]
6: child drank ORS	38	41	92.7	14	14	100.0	24	27	88.9	-	0.54[a]
7: child drank recommended fluids	31	38	81.6	12	14	85.7	19	24	79.2	-	1.00[a]
8: child drank other fluids	29	31	93.5	12	12	100.0	17	19	89.5	-	0.51[a]
9: how much did child drank during diarrhoea	27	29	93.1	11	12	91.7	16	17	94.1	-	1.00[a]
10: child had been introduced to complementary food	23	27	85.2	8	11	72.7	15	16	93.8	-	0.27[a]
10a: how much child ate during diarrhoea	10	13	76.9	4	5	80.0	6	8	75.0	-	1.00[a]
11: sought care for	18	20	90.0	7	7	100.0	11	13	84.6	-	0.52[a]

diarrhoea										
12: why no care was sought for diarrhoea	7	7	100.0	2	2	100.0	5	5	100.0	-	-[b]
13: where care was sought for diarrhoea	9	11	81.8	5	5	100.0	4	6	66.7	-	0.45[a]
14: child had fever	474	510	92.9	167	182	91.8	307	328	93.6	0.60	0.44
15: child had illness with cough	433	474	91.4	159	167	95.2	274	307	89.3	4.86	0.03[c]
16: child breathed fast or with difficulty	398	433	91.9	149	159	93.7	249	274	90.9	1.09	0.30
17: cause of fast or difficult breathing	11	13	84.6	5	6	83.3	6	7	85.7	-	1.00[a]
18: sought care for the child during fever or cough	95	110	86.4	37	39	94.8	58	71	81.7	3.88	0.049[c]
19: why no care was sought for fever or cough	4	10	40.0	2	5	40.0	2	5	40.0	-	1.00[a]
20: where care was sought for fever or cough	66	85	77.6	26	32	81.3	40	53	75.5	0.48	0.49
Completion[d]	356	651	54.7	137	229	59.8	219	422	51.9	3.77	0.05

[a]Fisher’s exact test.

[b]No statistics could be calculated.

[c]*P*<0.05

[d]Completed all questions participants were supposed to complete; calculated based on numbers in Table S4.

Table S8. Comparison of conditions of children and responses between group 1 and group 2

Status number	Conditions[a]		Total group (N=651)	Group 1 (n=229)	Group 2 (n=422)
	-Diarrhoea (D)<br>-Complementary feeding (CF)	-Cough (C)<br>-Fever (F)<br>-Fast or difficult breathing (B)	n respond to all questions	n respond to all questions	n respond to all questions
1	None	None	276	103	173
2	None	F	12	5	7
3	None	C	35	13	22
4	None	B	4	2	2
5	None	C and F	17	7	10
6	None	C and B	4	2	2
7	None	F and B	0	0	0
8	None	C, F and B	2	1	1
9	D, CF	None	1	1	0
10	D, CF	F	0	0	0
11	D, CF	C	0	0	0
12	D, CF	B	0	0	0
13	D, CF	C and F	0	0	0
14	D, CF	C and B	0	0	0
15	D, CF	F and B	0	0	0
16	D, CF	C, F and B	0	0	0
17	D	None	5	3	2
18	D	F	0	0	0
19	D	C	0	0	0
20	D	B	0	0	0
21	D	C and F	0	0	0
22	D	C and B	0	0	0
23	D	F and B	0	0	0
24	D	C, F and B	0	0	0
Completion[b]			356	137	219

[a]The response rate per status could not be calculated for separate statuses, because it was not known to which status non-responders belonged.
[b]All statuses combined; participants who responded to all asked text message questions.

Table S9. Characteristics of responders versus non-responders in group 1

Variables	Total (N=371)	Did not respond (n=138)	Respond (n=233)	Comparison Statistics[a]	*P* value
**Gender, n (%)**				χ^2=0.70	0.40
Boy	204 (55.0)	72 (52.2)	132 (56.7)		
Girl	167 (45.0)	66 (47.8)	101 (43.3)		
**Age child groups, n (%)**				MWU/WW z=-0.28	0.78
0-11 months	74 (20.0)	29 (21.0)	45 (19.3)		
12-23 months	112 (30.2)	41 (29.7)	71 (30.5)		
24-59 months	185 (49.8)	68 (49.3)	117 (50.2)		
**Number of children, n (%)**				MWU/WW z=-1.87	0.06
1	174 (46.9)	57 (41.3)	117 (50.2)		
2	192 (51.8)	77 (55.8)	115 (49.4)		
3	4 (1.1)	3 (2.2)	1 (0.4)		
4	1 (0.2)	1 (0.7)	0 (0.0)		
**Mother's age in years, median (Q1-Q3)**	28 (26-31)	29 (27-32)	28 (26-31)	MWU/WW z=-1.09	0.27
**Mother's education level, median (Q1-Q3)[b]**	3 (3-4)	3 (3-4)	3 (3-4)	MWU/WW z=-0.11	0.91
**Mother's number of years of education, median (Q1-Q3)**	9 (9-11)	9 (9-12)	9 (9-11)	MWU/WW z=-0.38	0.70
**Mother's occupation, n (%)**				Fisher's exact Test	0.11
Home	171 (46.1)	70 (50.7)	101 (43.4)		
Work	199 (53.6)	67 (48.6)	132 (56.6)		
Do not know	1 (0.3)	1 (0.7)	0 (0.0)		
**Father's age in years, median (Q1-Q3)**	29 (27-32)	29 (27-32)	29 (27-31)	MWU/WW z=-1.46	0.14
**Father's education level, median (Q1-Q3)[b]**	3 (3-4)	3 (3-4)	3 (3-4)	MWU/WW z=-0.25	0.80
**Father's number of years of education, median (Q1-Q3)**	9 (9-12)	9 (9-11)	9 (9-12)	MWU/WW z=-0.10	0.92
**Father's occupation, n (%)**				Fisher's exact Test	0.05
Home	6 (1.6)	4 (2.9)	2 (0.9)		
Work	363 (97.8)	132 (95.7)	231 (99.1)		
Do not know	2 (0.6)	2 (1.4)	0 (0.0)		
**Relation to the child, n (%)**				Fisher's exact Test	0.06
Mother	300 (80.9)	120 (87.0)	180 (77.3)		
Father	58 (15.6)	17 (12.3)	41 (17.6)		
Grandmother	9 (2.4)	1 (0.7)	8 (3.4)		
Grandfather	4 (1.1)	0 (0.0)	4 (1.7)		
**Participant is primary caregiver, n (%)**				χ^2=2.39	0.12
Yes	279 (75.2)	110 (79.7)	169 (72.5)		
No	92 (24.8)	28 (20.3)	64 (27.5)		
**Registered as urban or rural, n (%)**				χ^2=8.10	0.004[c]
Urban	34 (9.2)	5 (3.6)	29 (12.5)		
Rural	337 (90.8)	133 (96.4)	204 (87.5)		

**Family net income in last year in ¥, median (Q1-Q3)**	20,000 (15,000-35,000)	20,000 (10,000-30,000)	24,500 (15,000-40,000)	MWU/WW z=-1.18	0.24
**Family living expenses in the last year in ¥, median (Q1-Q3)**	20,000 (10,000-20,000)	15,000 (10,000-20,000)	20,000 (10,000-20,000)	MWU/WW z=-1.15	0.25
**Use smartphone, n (%)**				χ^2=0.007	0.98
Yes	167 (45.0)	62 (44.9)	105 (45.1)		
No	204 (55.0)	76 (55.1)	128 (54.9)		
**Primary usage of mobile phone for calls and text messages, n (%)**				Fisher's exact test	0.54
Calling	297 (80.1)	114 (82.6)	183 (78.5)		
Text messaging	10 (2.7)	2 (1.4)	8 (3.4)		
Both in equal measure	62 (16.7)	22 (16.0)	40 (17.2)		
Other	2 (0.5)	0 (0.0)	2 (0.9)		
**Primary usage of mobile phone for *QQ* and text messages, n (%)**				Fisher's exact test	0.61
Text message	124 (33.4)	40 (29.0)	84 (36.1)		
QQ	147 (39.6)	57 (41.3)	90 (38.6)		
Both in equal measure	22 (5.9)	9 (6.5)	13 (5.6)		
Do not use either	77 (20.8)	32 (23.2)	45 (19.3)		
Other (seldom use)	1 (0.3)	0 (0.0)	1 (0.4)		
**Number of calls made per week, median (Q1-Q3)**	10 (7-21)	10 (5-20)	13.5 (7-21)	MWU/WW z=-1.89	0.06
**Number of calls received per week, median (Q1-Q3)**	10 (6-21)	10 (6-20)	11.5 (7-21)	MWU/WW z=-1.22	0.22
**Number of text messages sent per week, median (Q1-Q3)**	1 (0-7)	1 (0-5)	2 (0-7)	MWU/WW z=-1.58	0.11
**Number of text messages received per week, median (Q1-Q3)**	8 (5-15)	7 (4-14)	10 (5-15)	MWU/WW z=-1.99	0.046[c]
**Have phone number of health facility, n (%)**				χ^2=3.40	0.07
Yes	245 (66.0)	83 (60.1)	162 (69.5)		
No	126 (34.0)	55 (39.9)	71 (30.5)		
**Number of times using mobile phone to obtain health information in past 3 months, median (Q1-Q3)**	1 (1-3)	1 (1-3)	1 (1-3)	MWU/WW z=-0.32	0.75
**Child had diarrhoea, n (%)**				Fisher's exact test	0.06
Yes	21 (5.7)	4 (2.9)	17 (7.3)		
No	349 (94.1)	133 (96.4)	216 (92.7)		
Not known	1 (0.2)	1 (0.7)	0 (0.0)		
**Sought care for diarrhoea, n (%)**				Fisher's exact test	0.24
Sought care	19 (5.1)	4 (2.9)	15 (6.4)		
Did not seek care	2 (0.5)	0 (0.0)	2 (0.9)		
No diarrhoea	349 (94.4)	133 (97.1)	216 (92.7)		

**Child had fever, n (%)**				Fisher's exact test	0.60
Yes	34 (9.2)	10 (7.3)	24 (10.3)		
No	336 (90.6)	128 (92.7)	208 (89.3)		
Not known	1 (0.2)	0 (0.0)	1 (0.4)		
**Child had cough, n (%)**				χ^2=1.69	0.19
Yes	92 (24.8)	29 (21.0)	63 (27.0)		
No	279 (75.2)	109 (79.0)	170 (73.0)		
**Sought care for fever or cough, n (%)**				Fisher's exact test	0.14
Sought care	88 (23.7)	29 (21.0)	59 (25.3)		
Did not seek care	13 (3.5)	2 (1.5)	11 (4.7)		
No fever or cough	270 (72.8)	107 (77.5)	163 (70.0)		
**Child breathed fast or with difficulty, n (%)**				χ^2=0.46	0.50
Yes	17 (4.6)	5 (3.6)	12 (5.2)		
No	354 (95.4)	133 (96.4)	221 (94.8)		

[a]Chi-square (χ^2), Mann-Whitney U/ Wilcoxon W (MWU/WW), quartile (Q), z-score (z).
[b]3=junior high school, 4=senior high school/technical school.
[c]$p<0.05$

Table S10. Characteristics of completers versus non-completers in group 1

Variables	Total (N=371)	Did not completed (n=234)	Completed (n=137)	Comparison Statistics[a]	*P* value
**Gender, n (%)**				χ^2=0.13	0.72
Boy	204 (55.0)	127 (54.3)	77 (56.2)		
Girl	167 (45.0)	107 (45.7)	60 (43.8)		
**Age child groups, n (%)**				MWU/WW z=0.71	0.48
0-11 months	74 (20.0)	51 (21.8)	23 (16.8)		
12-23 months	112 (30.2)	68 (29.1)	44 (32.1)		
24-59 months	185 (49.8)	115 (49.1)	70 (51.1)		
**Number of children, n (%)**				MWU/WW z=-1.10	0.27
1	174 (46.9)	105 (44.9)	69 (50.4)		
2	192 (51.8)	125 (53.4)	67 (48.9)		
3	4 (1.1)	3 (1.3)	1 (0.7)		
4	1 (0.2)	1 (0.4)	0 (0.0)		
**Mother's age in years, median (Q1-Q3)**	28.0 (26.0-31.0)	29.0 (26.0-31.0)	28.0 (26.0-31.0)	MWU/WW z=-0.99	0.32
**Mother's education level, median (Q1-Q3)[b]**	3 (3-4)	3 (3-4)	3 (3-4)	MWU/WW z=-0.48	0.63
**Mother's number of years of education, median (Q1-Q3)**	9.0 (9.0-11.0)	9.0 (9.0-11.0)	9.0 (9.0-11.0)	MWU/WW z=-1.00	0.32
**Mother's occupation, n (%)**				Fisher's exact test	0.27
Home	171 (46.1)	114 (48.7)	57 (41.6)		
Work	199 (53.6)	119 (50.9)	80 (58.4)		
Do not know	1 (0.3)	1 (0.4)	0 (0.00)		
**Father's age in years, median (Q1-Q3)**	29.0 (27.0-32.0)	29.0 (27.0-32.0)	29.0 (26.0-31.0)	MWU/WW z=-0.92	0.36
**Father's education level, median (Q1-Q3)[b]**	3 (3-4)	3 (3-3)	3 (3-4)	MWU/WW z=1.58	0.11
**Father's number of years of education, median (Q1-Q3)**	9.0 (9.0-12.0)	9.0 (9.0-11.0)	9.0 (9.0-12.0)	MWU/WW z=0.45	0.65
**Father's occupation, n (%)**				Fisher's exact test	0.39
Home	6 (1.6)	5 (2.1)	1 (0.7)		
Work	363 (97.8)	227 (97.0)	136 (99.3)		
Do not know	2 (0.6)	2 (0.9)	0 (0.0)		
**Relationship to the child, n (%)**				Fisher's exact test	0.78
Mother	300 (80.9)	192 (82.1)	108 (78.8)		
Father	58 (15.6)	34 (14.5)	24 (17.5)		
Grandmother	9 (2.4)	6 (2.6)	3 (2.2)		
Grandfather	4 (1.1)	2 (0.8)	2 (1.5)		
**Participant is primary caregiver, n (%)**				χ^2=3.06	0.08
Yes	279 (75.2)	183 (78.2)	96 (70.1)		
No	92 (24.8)	51 (21.8)	41 (29.9)		

**Registered as urban or rural, n (%)**				χ^2=0.83	0.36
Urban	34 (9.2)	19 (8.1)	15 (11.0)		
Rural	337 (90.8)	215 (91.9)	122 (89.0)		
**Family net income in last year in ¥, median (Q1-Q3)**	20,000 (15,000-35,000)	20,000 (15,000-35,000)	30,000 (15,000-40,000)	MWU/WW z=1.07	0.28
**Family living expenses in the last year in ¥, median (Q1-Q3)**	20,000 (10,000-20,000)	20,000 (10,000-20,000)	20,000 (10,000-20,000)	MWU/WW z=-0.01	1.00
**Use smartphone, n (%)**				χ^2=0.25	0.61
Yes	167 (45.0)	103 (44.0)	64 (46.7)		
No	204 (55.0)	131 (56.0)	73 (53.3)		
**Primary usage of mobile phone for calls and text messages, n (%)**				Fisher's exact test	0.49
Calling	297 (80.1)	183 (78.2)	114 (83.2)		
Text messaging	10 (2.7)	8 (3.4)	2 (1.5)		
Both in equal measure	62 (16.7)	42 (18.0)	20 (14.6)		
Other	2 (0.5)	1 (0.4)	1 (0.7)		
**Primary usage of mobile phone for *QQ* and text messages, n (%)**				Fisher's exact test	0.16
Text message	124 (33.4)	69 (29.5)	55 (40.2)		
QQ	147 (39.6)	98 (41.9)	49 (35.8)		
Both in equal measure	22 (5.9)	15 (6.4)	7 (5.1)		
Do not use either	77 (20.8)	52 (22.2)	25 (18.3)		
Other (seldom use)	1 (0.3)	0 (0.0)	1 (0.6)		
**Number of calls made per week, median (Q1-Q3)**	10 (7-21)	10 (6-20)	14 (7-28)	MWU/WW z=1.49	0.14
**Number of calls received per week, median (Q1-Q3)**	10 (6-21)	10 (5.5-20)	10 (7-21)	MWU/WW z=0.88	0.38
**Number of text messages sent per week, median (Q1-Q3)**	1 (0-7)	2 (0-7)	1 (0-5)	MWU/WW z=-0.82	0.41
**Number of text messages received per week, median (Q1-Q3)**	8 (5-15)	7 (5-15)	10 (5-14)	MWU/WW z=1.12	0.26
**Have phone number of health facility, n (%)**				χ^2=1.06	0.30
Yes	245 (66.0)	150 (64.1)	95 (69.3)		
No	126 (34.0)	84 (35.9)	42 (30.7)		
**Number of times using mobile phone to obtain health information in past 3 months, median (Q1-Q3)**	1 (1-3)	1 (1-3)	1 (1-3)	MWU/WW z=-0.65	0.52

**Child had diarrhoea, n (%)**				Fisher's exact test	0.03[c]
Yes	21 (5.7)	18 (7.7)	3 (2.2)		
No	349 (94.0)	215 (91.9)	134 (97.8)		
Not known	1 (0.3)	1 (0.4)	0 (0.00)		
**Sought care for diarrhoea, n (%)**				Fisher's exact test	0.06
Sought care	19 (5.1)	16 (6.9)	3 (2.2)		
Did not seek care	2 (0.5)	2 (0.9)	0 (0.0)		
No diarrhoea	349 (94.4)	215 (92.2)	134 (97.8)		
**Child had fever, n (%)**				Fisher's exact test	0.48
Yes	34 (9.2)	21 (9.0)	13 (9.5)		
No	336 (90.6)	213 (91.0)	123 (89.8)		
Not known	1 (0.2)	0 (0.0)	1 (0.7)		
**Child had cough, n (%)**				χ^2=0.26	0.61
Yes	92 (24.8)	56 (23.9)	36 (26.3)		
No	279 (75.2)	178 (76.1)	101 (73.7)		
**Sought care for fever or cough, n (%)**				Fisher's exact test	0.89
Sought care	88 (23.7)	54 (23.1)	34 (24.8)		
Did not seek care	13 (3.5)	8 (3.4)	5 (3.7)		
No fever or cough	270 (72.8)	172 (73.5)	98 (71.5)		
**Child breathed fast or with difficulty, n (%)**				χ^2=0.14	0.71
Yes	17 (4.6)	10 (4.3)	7 (5.1)		
No	354 (95.4)	224 (95.7)	130 (94.9)		

[a]Chi-square (χ^2), Mann-Whitney U/ Wilcoxon W (MWU/WW), z-score (z).
[b]3=junior high school, 4=senior high school/technical school.
[c]$p<0.05$

Table S11. Face-to-face versus text message answers to nominal dichotomous questions (N=409)

**Face-to-face**[a]	**Text message**		
**Text message: brief content**	**Yes**	**No**	**Do not know**
**4: child had diarrhoea (n=409)**			
Yes	23	2	0
No	9	373	1
Do not know	0	1	0
**5: child had blood in stools (n=21)**			
Yes	0	0	0
No	0	21	0
Do not know	0	0	0
**6: child drank ORS (n=19)**			
Yes	1	2	0
No	1	15	0
Do not know	0	0	0
**7: child drank recommended fluids (n=16)**			
Yes	14	2	0
No	0	0	0
Do not know	0	0	0
**8: child drank other fluids (n=16)**			
Yes	5	0	0
No	2	9	0
Do not know	0	0	0
**11: sought care for diarrhoea (n=10)**			
Yes	5	1	0
No	2	2	0
Do not know	0	0	0
**14: child had fever (n=365)**			
Yes	25	7	0
No	10	322	0
Do not know	1	0	0
**15: child had illness with cough (n=332)**			
Yes	62	19	0
No	18	233	0
Do not know	0	0	0
**16: child breathed fast or with difficulty (n=308)**			
Yes	9	6	0
No	2	290	0
Do not know	0	1	0
**18: sought care for child during fever or cough (n=55)**			
Yes	46	2	0
No	1	6	0
Do not know	0	0	0

[a]Grey indicates answers that were the same when comparing the face-to-face and text messaging survey; black indicates answers that were different when comparing the face-to-face and text messaging survey.

Table S12. Face-to-face versus text message answers to nominal non-dichotomous question about cause of fast or difficult breathing (N=409)

Face-to-face[a]	Text message			
**Text message: brief content**	**Problem in chest**	**Blocked nose**	**Both**	**Other**
**17: cause of breathing fast or difficult (n=8)**				
Problem in chest	2	0	1	0
Blocked nose	0	4	0	0
Both	0	0	0	0
Other[*]	1	0	0	0

[a]Bronchitis.

Table S13. Face-to-face versus text message answers to nominal non-dichotomous questions about reason for not seeking care (N=409)

Face-to-face	Text message
**Text message: brief content**	**Mild disease**
**12: why care was not sought for diarrhoea (n=2)**	
Mild disease	2
**19: why care was not sought for fever or cough (n=2)**	
Mild disease	2

Table S14. Face-to-face versus text message answers to nominal non-dichotomous question about where participants sought care for diarrhoea (N=409)

Face-to-face	Text message	
**Text message: brief content**	**Village clinic**	**Village clinic, county level hospital or above[*], county-level Maternal and Child Health Hospital**
**13: where sought care for diarrhoea (n=4)**		
Village clinic	3	0
Village clinic, county level hospital or above[a], county-level Maternal and Child Health Hospital	1	0

[a]Excluding county level Maternal and Child Health Hospital.

Table S15. Face-to-face versus text message answers to nominal non-dichotomous question about where participants sought care for fever or cough (N=409)

Face-to-face[a]	Text message											
**Text message: brief content**	**1**	**2**	**3**	**4**	**5**	**6**	**1+4**	**1+6**	**2+6**	**4+5**	**4+6**	**4+7**
**20: where care was sought for fever or cough (n=40)**												
0	0	0	1	1	0	0	0	0	0	0	0	1
1	2	0	0	0	0	0	0	0	0	0	0	0
2	0	0	0	0	0	0	0	0	1	0	0	0
3	0	0	0	0	0	0	0	0	0	0	0	0
4	0	0	0	16	0	2	0	0	0	1	1	0
5	0	0	0	0	1	1	0	0	0	0	0	0
6	0	0	0	1	0	4	0	1	0	0	0	0
7	0	0	0	0	0	0	0	0	0	0	0	0
1+2	0	1	0	0	0	0	0	0	0	0	0	0
1+4	0	0	0	0	0	0	1	0	0	0	0	0
2+6	0	0	0	0	0	0	0	0	1	0	0	0
3+4	0	0	0	0	0	0	0	0	0	0	0	0
4+6	0	0	0	0	0	0	0	0	0	0	1	0
4+8	0	0	0	1	0	0	0	0	0	0	0	0
1+2+6	0	0	0	0	0	1	0	0	0	0	0	0

[a]Places where care was sought were listed as follows:
0= no places reported; participants said "no" for all the places where care was sought and no place was recorded in the face-to-face interview;
1= County level hospital or above (excluding Maternal and Child Health Hospital);
2= County level Maternal and Child Health Hospital;
3= Township hospital;
4= Village clinic;
5= Private hospital;
6= Private clinic;
7= Pharmacy;
8= Community health station.

Table S16. Face-to-face versus text message answers to ordinal questions (N=409)

**Face-to-face**	**Text message**					
**Text message: brief content**	**None**	**Much less**	**Little less**	**About the same**	**More**	**No foods yet**
**9: how much child drank during diarrhoea (n=14)**						
None	0	0	0	0	0	NA
Much less	0	2	0	0	0	
Little less	0	0	0	0	0	
About the same	1	0	1	9	0	
More	0	0	0	0	1	
**10: how much child ate during diarrhoea (n=11)**						
None	0	0	0	0	0	1
Much less	0	2	0	1	0	0
Little less	0	0	1	0	0	0
About the same	0	0	0	0	1	3
More	0	0	0	0	1	0
No foods yet	0	0	0	0	0	1

Table S17. Face-to-face versus text message answers to question about being introduced to complementary foods (N=409)

**Face-to-face**	**Text message**	
**Text message: brief content**	Yes	No
**10a: introduced to complementary food (n=12)[a]**		
Yes	7	4
No	0	1

[a]One participant said that the child was introduced to complementary food in the text messaging survey, but did not respond to the next question in text message 10 about how much food was given. Therefore, the number of participants is 12 and not 11 as indicated for text message 10 in Table 16.

Table S18. Face-to-face versus text message number of places participants reported for question about seeking care for diarrhoea (N=409)

Number of face-to-face responses	Number (n) of text message responses		
**Text message: brief content**	**1**	**2**	**3**
**13: where care was sought for diarrhoea (n=4)**			
1	3	0	0
2	0	0	0
3	1	0	0

Table S19. Face-to-face versus text message number of places participants reported for question about seeking care for fever or cough (N=409)

Number of face-to-face responses	Number (n) of text message responses		
**Text message: brief content**	**1**	**2**	**3**
**20: where care was sought for fever or cough (n=40)**			
0	2	1	0
1	27	4	0
2	2	3	0
3	1	0	0

Table S20. Participants' reasons for different responses in group 2 (n=226)

			Text message[c]						Face-to-face[d]					Other[e]	
**Text message: brief content**	**n[a]**	**Missing[b]**	**A**	**B**	**C**	**D**	**E**	**F**	**G**	**H**	**I**	**J**	**K**	**L**	**M**
4: child had diarrhoea	12	2	2	2	1		1	1	1	2					
5: child had blood in stools	0														
6: child drank ORS	2	1								1					
7: child drank recommended fluids	0														
8: child drank other fluids	0														
9: how much child drank during diarrhoea	0														
10a: how much child ate during diarrhoea	4	2		1						1					
11: sought care for diarrhoea	2	2													
12: why no care was sought for diarrhoea	1	1													
13: where care was sought for diarrhoea	0														
14: child had fever	10		3		1	1			1	1	1	1		1	
15: child had illness with cough	16		3		1				5	1	2	1	1	1	1
16: child breathed fast or with difficulty	5	1							1	1	1				
17: cause of fast or difficult breathing	0														
18: sought care for child during fever or cough	0														
19: why no care was sought for fever or cough	0														
20: where care was sought for fever or cough	0														
Total	51	9	8	3	3	1	1	1	8	7	4	2	1	2	1

[a]Number of the same caregivers responding to both text message and face-to-face question and giving a different response.
[b]Missing because forgot to ask (text message 4, 6, 10a, 11, 16) or error in text message answer (question 12).
[c] <u>text message related reasons:</u>
A= did not see the date (there were dates in the text messages to ask about the past two weeks);
B= misunderstood question;
C= replied carelessly and did not pay attention;
D= put wrong answer by mistake;
E= forgot how reply was given;
F= did not see the accurate definition of diarrhoea.
[d]<u>face-to-face related reasons:</u>
G= did not know the accurate definition of a symptom, diarrhoea (number 4) the temperature of fever (number 14), cough caused by illness (number 15), fast and difficult breathing (number 16);
H= misunderstood question;
I= did not understand the date clearly;
J= did not hear the question clearly;
K= interviewer was in a hurry.
[e]<u>other reasons (not mentioned to be related to text message or face-to-face method):</u>
L= could not recall the requested information for the question;
M= changed mind.

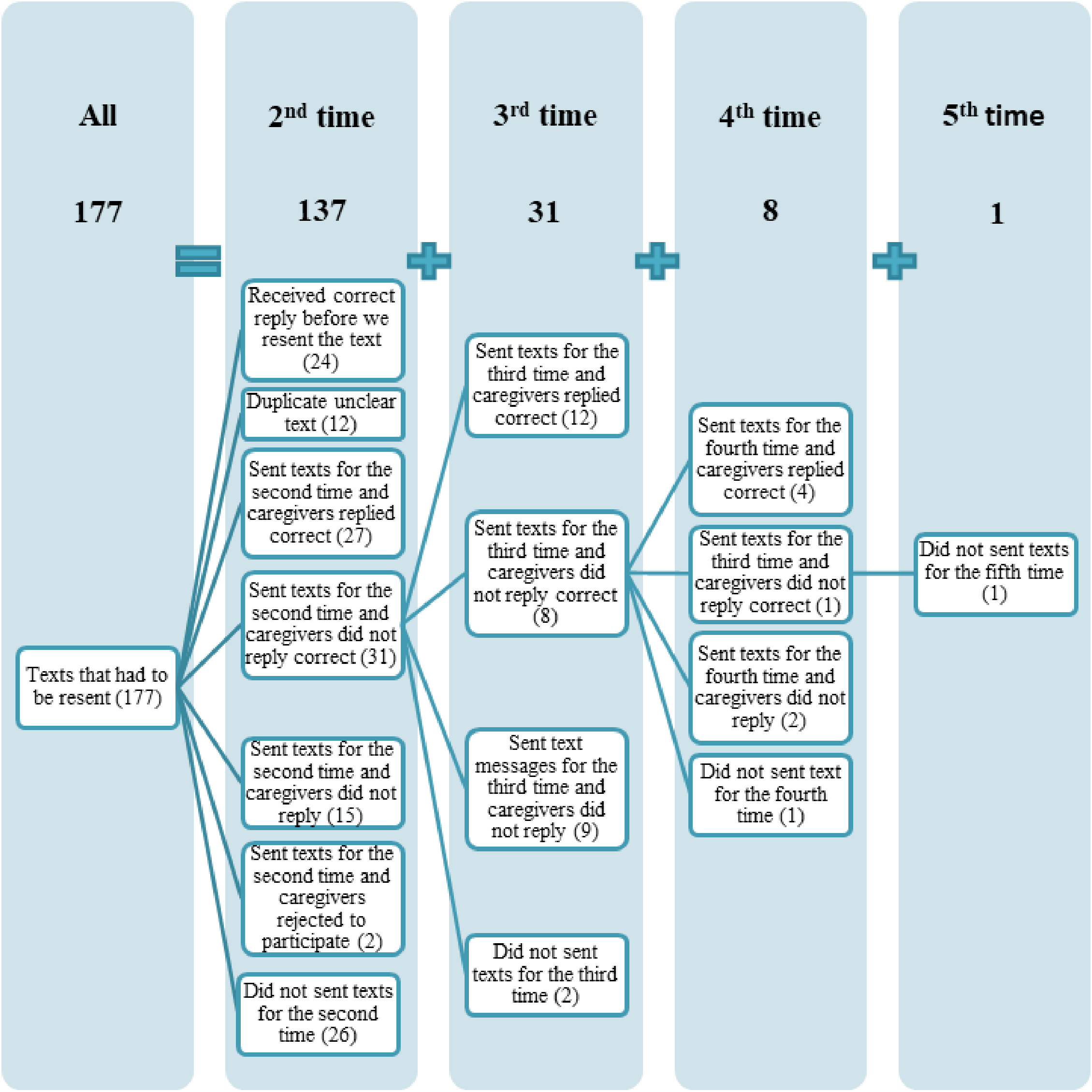

Figure S1 Unclear text messages that had to be resent
